# Circulating Tumor DNA for Minimal Residual Disease Detection and Recurrence Prediction in Upper Gastrointestinal Cancers: A Scoping Review

**DOI:** 10.3390/jcm15166222

**Published:** 2026-08-11

**Authors:** Loizos Hadjigeorgiou, Melina Yerolatsite, Nanteznta Torounidou, George Zarkavelis, Dimitrios Schizas, Vasileios Tatsis, Stefano Rausei, Konstantinos Vlachos, Georgios D. Lianos

**Affiliations:** 1 Department of General Surgery, University Hospital of Ioannina, 45500 Ioannina, Greece; tatsis.vasileios@hotmail.com (V.T.); kvlachos@uoi.gr (K.V.); glianos@uoi.gr (G.D.L.); 2 Department of Medical Oncology, University Hospital of Ioannina, 45500 Ioannina, Greece; m.yerolatsite@gmail.com (M.Y.); nadia.torou@gmail.com (N.T.); gzarkavelis@outlook.com (G.Z.); 3 First Department of Surgery, Laikon General Hospital, National and Kapodistrian University of Athens, 11527 Athens, Greece; schizasad@gmail.com; 4 General Surgery Unit, Department of Surgery, Cittiglio-Angera Hospital, ASST Sette Laghi, 21100 Varese, Italy; stefano.rausei@gmail.com

**Keywords:** circulating tumor DNA, ctDNA, minimal residual disease, liquid biopsy, esophageal cancer, gastric cancer, recurrence, prognosis

## Abstract

Circulating tumor DNA (ctDNA) is a promising non-invasive biomarker for detecting minimal residual disease (MRD) and predicting recurrence after curative treatment, yet evidence in esophageal squamous cell carcinoma (ESCC), esophageal adenocarcinoma (EAC), and gastric cancer has largely been examined within individual tumor types. In this scoping review, we mapped this evidence across all three malignancies and clarified key methodological and clinical considerations. Following the PRISMA-ScR guidelines, we searched PubMed, Scopus, and the Cochrane Library (3 April 2026) for studies linking ctDNA to disease-free, recurrence-free, or overall survival after curative-intent treatment. Twenty-seven studies (1746 patients; 10 ESCC, 4 EAC, 8 gastric, and 5 mixed) were included. Across every tumor type, postoperative ctDNA MRD was the most informative timepoint, with independent multivariable hazard ratios for disease-free, recurrence-free, or event-free survival of 2.8 to 21.8, whereas preoperative ctDNA was seldom prognostic. Serial monitoring further improved performance and flagged recurrence 78 to 278 days before imaging. Tumor-informed assays showed higher sensitivity than tumor-agnostic ones (80% vs. 35%), though direct comparisons were limited; correction for clonal hematopoiesis was essential for tumor-agnostic assays, and blood-based assays performed poorly in diffuse-type and peritoneal disease. Postoperative ctDNA MRD is a consistent, independent prognostic biomarker across upper gastrointestinal cancers that adds prognostic information beyond conventional staging and pathological response, supporting prospective interventional trials of ctDNA-guided management.

## 1. Introduction

Upper gastrointestinal cancers—esophageal squamous cell carcinoma (ESCC), esophageal adenocarcinoma (EAC), and gastric cancer (GC)—are among the most lethal malignancies worldwide. In GLOBOCAN 2022, gastric cancer was the fifth most common cancer and the fifth leading cause of cancer death (~970,000 new cases and 660,000 deaths annually), while esophageal cancer ranked eleventh in incidence and seventh in mortality (>510,000 cases and 450,000 deaths) [1]. The histological balance shifts by region—ESCC predominates in East Asia and Sub-Saharan Africa, EAC in Western countries—yet both esophageal subtypes are biologically aggressive and present late, which largely explains their poor prognosis [2,3].

For localized disease, curative-intent multimodal therapy—neoadjuvant or perioperative chemotherapy or chemoradiotherapy combined with radical resection—offers the only realistic chance of cure, yet recurrence remains the central clinical problem. Despite advances such as FLOT-based perioperative chemotherapy for gastric and gastroesophageal junction cancer [4] (building on the MAGIC paradigm [5]) and CROSS-based neoadjuvant chemoradiotherapy for esophageal cancer [6,7], more than 60% of patients relapse after curative gastrectomy, about 42% within the first two years [8]; even after chemoradiotherapy and surgery, 5-year overall survival was only ~43% for esophageal and junctional adenocarcinoma in the landmark CROSS trial [6]. Most recurrences are diagnosed at an advanced or metastatic stage, when curative salvage is rarely possible—only 3.2% of patients with recurrent gastric cancer receive potentially curative re-treatment [9]—and survival thereafter is measured in months rather than years.

Postoperative surveillance relies mainly on scheduled cross-sectional imaging (typically, computed tomography every three to six months) and serum tumor markers such as carcinoembryonic antigen (CEA) and carbohydrate antigen 19-9 (CA19-9) [10,11]. Neither detects residual disease reliably at an early stage: CEA and CA19-9 have reported sensitivities of just 34% and 24%, respectively, for postoperative gastric cancer recurrence [12], and cross-sectional imaging has limited, variable sensitivity for early recurrence, performing especially poorly for peritoneal disease [13]. By the time recurrence becomes radiologically apparent, molecular disease has usually been present for months—a window in which earlier detection might allow intervention before the shift to widely metastatic disease.

Circulating tumor DNA (ctDNA)—tumor-derived DNA fragments released into the bloodstream through apoptosis, necrosis, and active secretion—has emerged as a promising non-invasive biomarker capable of addressing this gap [14,15,16]. As a fraction of total cell-free DNA (cfDNA), it carries tumor-specific somatic mutations, copy-number alterations, and epigenetic marks that distinguish it from non-tumor cfDNA. Mutation-based assays identify ctDNA directly through these tumor-specific variants, whereas methylation-based assays—which are typically tumor-agnostic—instead detect the tumor-derived fraction of cfDNA from cancer-specific methylation signatures; in the latter, ctDNA is quantified as an estimated tumor fraction of the total cfDNA pool rather than as a count of individual mutant molecules. Advances in next-generation sequencing—ultradeep hybrid-capture sequencing, tumor-informed multiplex PCR-based NGS (e.g., Signatera), and methylation-based platforms—have markedly improved sensitivity and specificity [17], enabling detection at variant allele fractions as low as 0.001% [18]. ctDNA detected after surgery, when resection should have cleared all macroscopic disease, is now regarded as a marker of minimal residual disease (MRD): occult micro-metastatic deposits that fall below imaging resolution yet carry a high risk of clinical recurrence.

The clinical value of ctDNA MRD is best established in colorectal cancer [19,20,21], where the randomized DYNAMIC trial showed that a ctDNA-guided approach to adjuvant therapy in stage II colon cancer reduced treatment without compromising recurrence-free survival [22]. Evidence in upper gastrointestinal cancers is also growing, with postoperative ctDNA positivity repeatedly linked to recurrence and worse survival, often several months before radiological detection. Yet the literature remains fragmented: existing systematic reviews and meta-analyses have examined individual tumor types in isolation—most notably esophageal [23,24] and gastric cancer [25]—and none has yet mapped ctDNA MRD evidence across the full spectrum of upper gastrointestinal malignancies within a single framework.

This fragmentation has clinical consequences. The three cancers sit close together anatomically, are treated similarly, and recur and are monitored in comparable ways, but they differ in their biology, in how much ctDNA they shed, and in where they tend to relapse. Mapping the evidence for all three together is, therefore, needed to separate the principles they share from the histotype-specific details that should shape ctDNA protocols and interventional trials.

This scoping review, therefore, maps the available evidence on ctDNA for MRD detection and recurrence prediction in patients with ESCC, EAC, and gastric cancer treated with curative intent—organized by tumor type, clinical context, and ctDNA platform—drawing out the key methodological and clinical considerations and identifying priorities for future research. It was conducted and reported in accordance with the PRISMA Extension for Scoping Reviews (PRISMA-ScR) [26].

## 2. Materials and Methods

### 2.1. Study Design

This scoping review was conducted following the framework of Arksey and O’Malley [27], as refined by Levac et al. [28], and reported in accordance with the PRISMA-ScR checklist [26]; the review was not registered in a public registry, and no protocol was published a priori. A scoping design was chosen because our aim was to map the breadth and characteristics of the evidence on circulating tumor DNA (ctDNA) as a biomarker for minimal residual disease (MRD) detection and recurrence prediction across all upper gastrointestinal (GI) cancers—esophageal squamous cell carcinoma (ESCC), esophageal adenocarcinoma (EAC), and gastric cancer (GC)—rather than to answer a single, narrowly defined question. The marked heterogeneity in study design, ctDNA platform, sampling timepoint, and outcome definition further favored a scoping review over a systematic review with meta-analysis.

### 2.2. Eligibility Criteria

Studies were included if they met all the following criteria:

- Population: Patients with histologically confirmed esophageal cancer (ESCC or EAC) or gastric cancer, with or without involvement of the gastroesophageal junction (GEJ), undergoing curative-intent treatment (surgery ± neoadjuvant or adjuvant therapy) or, where relevant, surveillance following resection.
- Intervention/Exposure: Measurement of ctDNA or cell-free DNA (cfDNA) in peripheral blood or peritoneal lavage fluid at one or more clinically relevant timepoints.
- Comparator: Studies were eligible regardless of whether a comparator group was present, given the exploratory nature of the scoping design.
- Outcomes: Studies reporting at least one of the following: recurrence rates stratified by ctDNA status; disease-free survival (DFS), recurrence-free survival (RFS), progression-free survival (PFS), or overall survival (OS) according to ctDNA status; sensitivity, specificity, or predictive values of ctDNA for recurrence detection; or lead time between ctDNA positivity and radiological or clinical confirmation of recurrence.
- Study design: Original research articles, including prospective and retrospective cohort studies, randomized controlled trial substudies, and proof-of-concept feasibility studies. Review articles, editorials, letters without original data, notes, tombstone publications, meeting or conference abstracts, book chapters, preprints, and case reports were excluded.
- Language: English-language publications only.
- Human studies only.

### 2.3. Search Strategy

We searched three electronic databases—PubMed, Scopus, and the Cochrane Library—on 3 April 2026. The strategy combined three thematic blocks with the Boolean operator AND (Table 1): Block 1, ctDNA and liquid biopsy terms; Block 2, recurrence and survival endpoints; and Block 3, tumor site. No date limits were applied at this stage, and searches were restricted to human studies published in English. Full search algorithms for each database appear in Supplementary File S1.

**Table 1:** Search strategy overview.

Database	Field Tags	Wildcard	Date Searched
PubMed	[MeSH], [tw], [tiab]	* (truncation)	3 April 2026
Scopus	TITLE-ABS-KEY ()	* (truncation)	3 April 2026
Cochrane Library	:ti,ab,kw + MeSH descriptors	* (truncation)	3 April 2026

**Figure 1:**
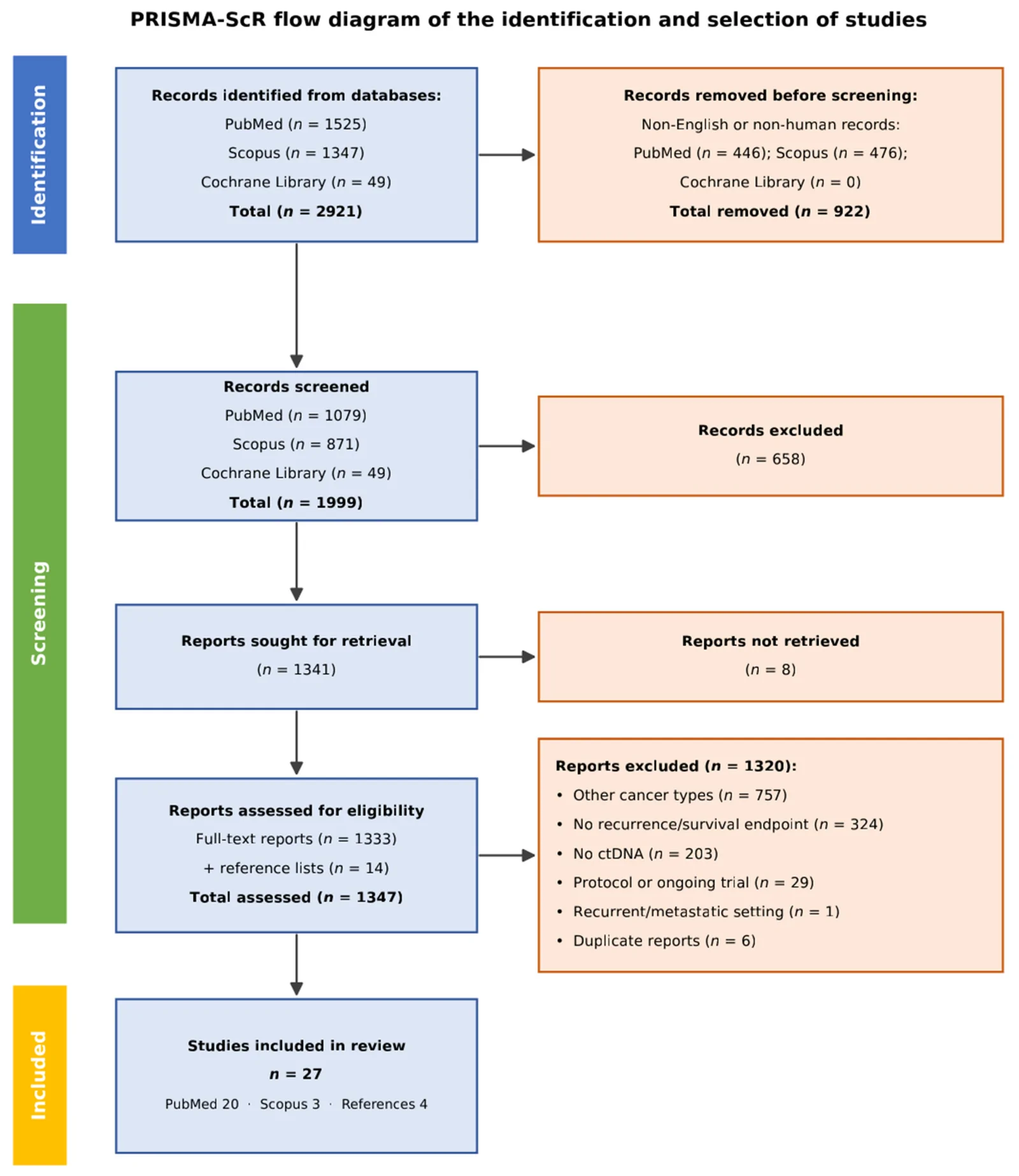
PRISMA-ScR flow diagram of the identification and selection of studies.

Human and English-language filters were applied at the database level where available, and the remaining non-English or non-human records that were nonetheless returned were removed before title-and-abstract screening (Figure 1). No date restrictions were applied in the search stage. The three thematic blocks were combined using the Boolean operator AND. Full search algorithms are provided in Supplementary File S1. Truncation (wildcard) symbol * used to capture all word-ending variants of a search term.

For Block 3 (tumor site), we deliberately searched the full spectrum of digestive system neoplasms rather than upper GI terms alone to maximize sensitivity and avoid missing relevant studies—consistent with scoping-review best practice [27,28]. This breadth captured mixed upper and lower GI cohorts and gastroesophageal junction (GEJ) tumors filed under varied site-specific headings that a narrow query would miss; screening then retained only ESCC, EAC, and gastric cancer studies. Favoring sensitivity over specificity in the search stage accords with PRISMA-ScR guidance, which notes that scoping searches may span broader topics than the final review scope [26].

### 2.4. Study Selection Process

All records retrieved from the three databases were imported into a reference management system and deduplicated. Two independent reviewers screened titles and abstracts against the predefined eligibility criteria; records that were clearly irrelevant were excluded in this stage. Full texts of potentially eligible studies were subsequently retrieved and assessed independently. Disagreements in any stage were resolved by discussion and consensus. The study selection process is presented in a PRISMA-ScR flow diagram (Figure 1).

Studies identified through the broad digestive system search that pertained exclusively to lower GI malignancies (colorectal cancer, anal cancer), hepatopancreaticobiliary tumors (hepatocellular carcinoma, cholangiocarcinoma, pancreatic cancer, gallbladder cancer), or small intestinal tumors were excluded at the full-text screening stage, as they fell outside the defined scope of this review.

### 2.5. Data Extraction

**Figure 2:**
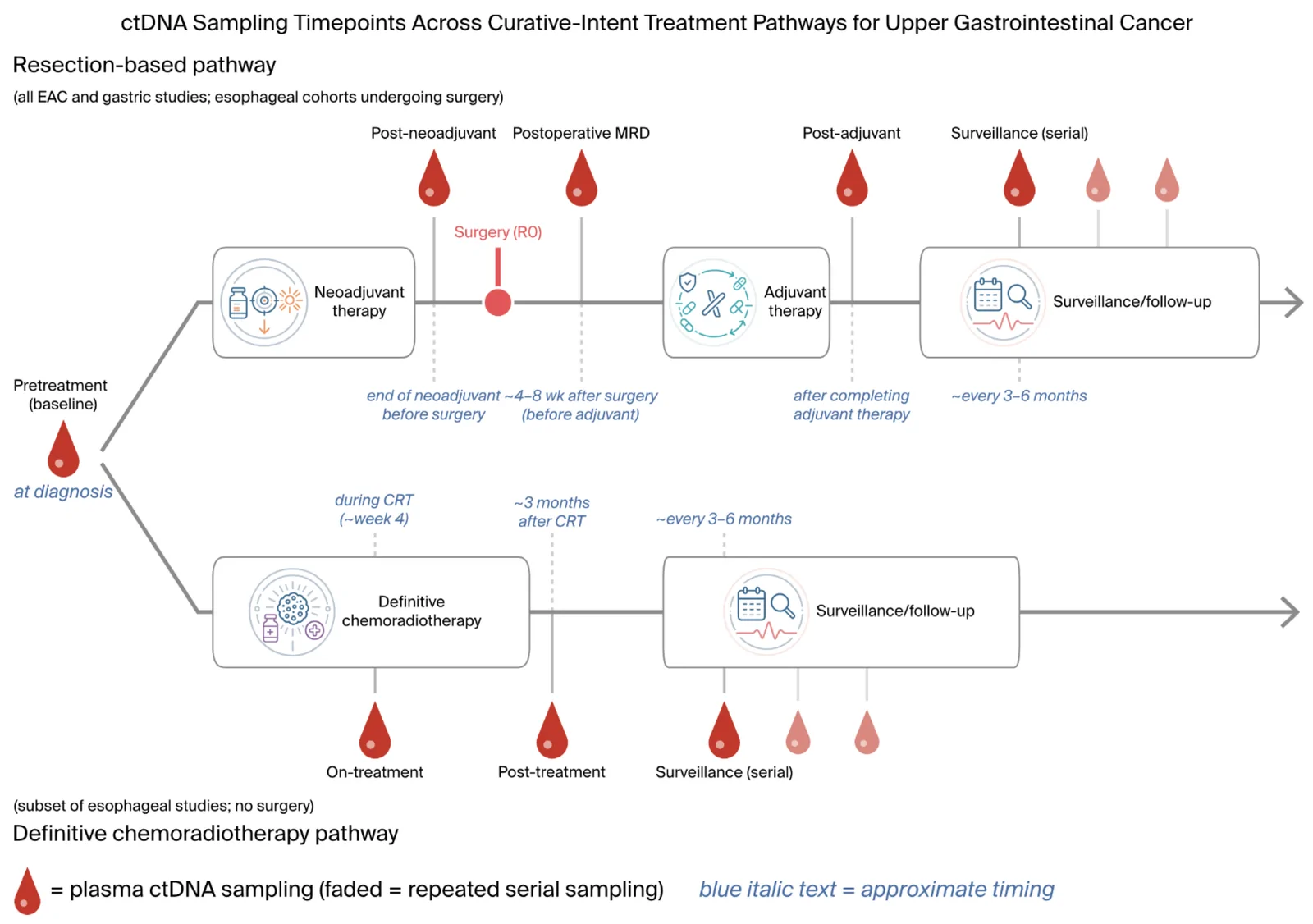
Schematic of circulating tumor DNA (ctDNA) sampling timepoints across the two curative-intent treatment pathways for upper gastrointestinal cancer, with the approximate timing of each sample. Most included studies followed a resection-based pathway (all esophageal adenocarcinoma and gastric studies, and esophageal squamous cell cohorts undergoing surgery), in which plasma ctDNA is sampled at pre-treatment (baseline, at diagnosis—baseline tumor burden), post-neoadjuvant (end of neoadjuvant therapy, before surgery—response to neoadjuvant treatment), postoperative minimal residual disease (MRD; approximately four to eight weeks after resection and before adjuvant therapy, with an observed range of roughly one to sixteen weeks across studies—residual disease and the principal prognostic landmark), post-adjuvant (after completion of adjuvant therapy—residual disease after all planned treatment), and surveillance (serial follow-up, approximately every three to six months—early recurrence detection and lead time). A subset of esophageal studies instead used definitive chemoradiotherapy without surgery, in which ctDNA is sampled at pre-treatment (baseline), on-treatment (during chemoradiotherapy, e.g., week 4), post-treatment (approximately three months after chemoradiotherapy—response to and residual disease after treatment), and surveillance. Faded droplets denote repeated serial sampling. MRD means minimal residual disease, and R0 means microscopically margin-negative resection.

Both reviewers extracted data independently using a standardized, pre-piloted form built in Microsoft Excel. For each study, we recorded: first author and year; country; design (prospective vs. retrospective; single-center vs. multi-center; randomized vs. non-randomized); number of patients; sex distribution and median age; tumor type and histological subtype; clinical and pathological staging; treatment received (neoadjuvant, adjuvant, or curative-intent surgery); ctDNA platform and methodology (tumor-informed vs. tumor-agnostic; sequencing technology; gene panel size; detection threshold); blood sampling timepoints; ctDNA positivity rates at each timepoint; and outcomes, including recurrence rates; DFS, RFS, PFS, and OS (as hazard ratios with 95% confidence intervals where available); sensitivity; specificity; positive and negative predictive values (PPVs, NPVs); and lead time to clinical or radiological recurrence. Discrepancies were resolved by discussion and by re-checking the original articles. Sampling timepoints were categorized as pre-treatment (baseline), post-neoadjuvant (after neoadjuvant therapy and before surgery), postoperative minimal residual disease (a landmark approximately four to eight weeks after resection), post-adjuvant (after completion of adjuvant therapy), and surveillance (serial sampling during follow-up), as each addresses a distinct clinical question. These timepoints, and the two curative-intent pathways in which they arise (surgical resection or, in a subset of esophageal studies, definitive chemoradiotherapy), are summarized schematically in Figure 2. For terminology, a detected postoperative signal is referred to as minimal residual disease (MRD)-positive, whereas a non-detected result is reported as “ctDNA not detected” (ctDNA-negative) rather than “MRD-negative”, because a negative plasma assay does not exclude residual disease present below the assay’s limit of detection.

### 2.6. Quality Assessment

Although a formal quality assessment is not required in scoping reviews [27,28], we appraised each included study using the Newcastle–Ottawa Scale (NOS) for cohort studies, adapted as needed for single-arm designs [29]. The NOS spans three domains—selection (up to 4 stars), comparability (up to 2 stars), and outcome ascertainment (up to 3 stars)—for a maximum of 9; we rated scores of 7–9 as high quality, 4–6 as moderate, and 0–3 as low. Both reviewers scored independently and resolved disagreements by consensus. Within the selection domain, one star each was awarded for a representative curative-intent cohort (a consecutive or complete series rather than a selected or availability-filtered subset), for drawing the ctDNA-negative comparison group from the same cohort and assay as the ctDNA-positive patients, for ascertainment of ctDNA status using a clearly described assay with a defined positivity threshold, and for demonstrating that patients were disease-free at the relevant baseline (typically an R0 resection with no radiographic disease at the minimal residual disease landmark). For comparability, the first star required that the association between ctDNA status and outcome be adjusted for tumor stage in a multivariable model (or by matching or stratification), and the second required that at least one further prognostic factor—such as adjuvant therapy, histology, or nodal status—also be included; studies reporting only univariable comparisons (log-rank or Fisher tests with unadjusted hazard ratios) scored zero in this domain. For outcome, one star each was awarded for objective recurrence ascertainment (protocol imaging or pathology), for a follow-up duration long enough to capture the early recurrences that predominate in upper gastrointestinal cancer (a median of approximately 18–24 months or clearly sufficient event capture), and for essentially complete follow-up. In feasibility and single-arm studies without a formal comparison group, the ctDNA-negative subset was treated as the internal comparator, and purely descriptive designs without any adjusted analysis were scored zero for comparability; such designs, together with short or unreported follow-up, account for the lower-scoring studies. Scores ranged from 2 to 9; 14 of 27 studies were rated high quality, 12 moderate, and one—a proof-of-concept methylation feasibility study—low (full results in Supplementary Table S1). Scores were used descriptively—to contextualize findings and flag potential bias—not as grounds for exclusion.

### 2.7. Data Synthesis

Because the included studies varied so widely in design, patient population, ctDNA methodology, and reported outcomes, we did not attempt a formal meta-analysis. Instead, the data were synthesized narratively and grouped by tumor type (ESCC, EAC, gastric cancer, and mixed upper GI cohorts) and by clinical context (upfront surgical cohorts, neoadjuvant and serial ctDNA cohorts, perioperative monitoring, and post-surgical surveillance).

### 2.8. Use of Generative AI

Generative AI (Claude, Anthropic; Claude Opus 4.8) was used for language assistance only (grammar, phrasing, and readability). It was not used for the study design, literature search, study selection, data extraction, quality assessment, or interpretation of findings, all of which were performed by the authors, who have reviewed and edited all AI-assisted text and take full responsibility for the content.

## 3. Results

### 3.1. Study Selection

The search on 3 April 2026 identified 2921 records—PubMed (*n* = 1525), Scopus (*n* = 1347), and the Cochrane Library (*n* = 49). After removing 922 non-English or non-human records (PubMed, *n* = 446; Scopus, *n* = 476; Cochrane, *n* = 0), 1999 remained for title and abstract screening.

Screening excluded a further 658 clearly irrelevant records, leaving 1341 reports for full-text retrieval; eight could not be obtained, and the remaining 1333 were assessed together with 14 reports identified by hand-searching reference lists (total 1347). At full-text review, reports were excluded for cancer types outside scope (*n* = 757), no recurrence or survival endpoint (*n* = 324), no ctDNA (*n* = 203), being protocols or ongoing trials without original data (*n* = 29), or evaluation restricted to the recurrent/metastatic setting rather than curative-intent MRD or recurrence prediction (*n* = 1). After six further duplicates were removed, 27 studies met all eligibility criteria and were included.

The full selection process is shown in Figure 1 (PRISMA-ScR flow diagram).

### 3.2. Characteristics of Included Studies

The 27 studies, published between 2019 and 2025, enrolled 1746 patients with upper gastrointestinal malignancies. Most originated from China (*n* = 15), with fewer from the United States (*n* = 2), the United Kingdom (*n* = 2), and France (*n* = 2); single studies from Belgium, the Netherlands, Denmark, Japan, and Korea; and one multinational collaboration (Netherlands/Sweden/Denmark). Twenty-one were prospective and six retrospective, the prospective studies comprising observational cohorts, randomized controlled trial substudies, and proof-of-concept feasibility studies. Four tumor groups were represented: esophageal squamous cell carcinoma (ESCC; *n* = 10), esophageal adenocarcinoma (EAC; *n* = 4), gastric cancer (GC; *n* = 8), and mixed upper gastrointestinal cohorts (*n* = 5). Detailed characteristics appear in Table 2a–d. Eligibility was determined by curative-intent treatment rather than by tumor stage. Accordingly, several included cohorts enrolled some patients with stage IV disease—including one small gastric series in which such patients predominated—who were nonetheless managed with curative intent, whether by definitive chemoradiotherapy for locally advanced esophageal cancer or by curative-intent gastrectomy; these studies were retained. By contrast, the single study restricted to ctDNA monitoring during palliative systemic therapy for established recurrent or metastatic disease did not meet this criterion and was excluded (Section 3.1).

The ten ESCC studies (Table 2a) were all from China, consistent with regional incidence; sample sizes ranged from 35 to 132 patients (median, 52.5), with seven prospective and three retrospective. Four studies enrolled patients undergoing curative-intent esophagectomy without neoadjuvant therapy, and six addressed neoadjuvant chemoradiotherapy (nCRT) followed by surgery (two randomized immunotherapy trials). Most patients had stage II–III disease; median follow-up was 12 to 60 months.

The four EAC studies (Table 2b) came from Belgium, the United Kingdom (two), and the Netherlands, mirroring the Western predominance of adenocarcinoma; sample sizes ranged from 20 to 97 patients, with three prospective and one retrospective. All four studies enrolled patients receiving neoadjuvant chemotherapy or chemoradiotherapy before curative esophagectomy, mostly cT3/T4, with a median follow-up of roughly 28 to 75 months.

The eight gastric cancer studies (Table 2c) were conducted in China (*n* = 5), France (*n* = 1), the Netherlands/Sweden/Denmark (*n* = 1), and Korea (*n* = 1), with sample sizes of 14 to 100 patients and an even prospective/retrospective split (4/4). All patients underwent curative-intent gastrectomy with or without perioperative chemotherapy; staging ranged from I to IV (mostly II–III), with a median follow-up of 12 to 52 months.

The five mixed upper gastrointestinal cohorts (Table 2d)—esophageal, gastroesophageal junction, and/or gastric cancer—were conducted in the United States (*n* = 2), France (*n* = 1), Denmark (*n* = 1), and Japan (*n* = 1), with sample sizes from 40 to 295 patients (the largest with 295 from more than 70 institutions); three were prospective and two retrospective. Treatment spanned perioperative chemotherapy and neoadjuvant chemoradiotherapy; median follow-up was approximately 14 to 29 months.

### 3.3. CtDNA Findings by Tumor Type

#### 3.3.1. Esophageal Squamous Cell Carcinoma (ESCC)

Ten studies examined ctDNA in ESCC, enrolling 636 patients in total across two clinical contexts: upfront surgical cohorts (*n* = 4) and neoadjuvant or serial ctDNA cohorts (*n* = 6). Key findings are summarized in Table 3a.

***Upfront surgical cohorts***

Four studies assessed ctDNA in ESCC patients undergoing curative esophagectomy without neoadjuvant therapy [30,31,32,39]. Preoperative positivity rates varied widely, from 54.3% [31] to 98.1% [32], reflecting differences in panel size, detection threshold, and staging. Preoperative ctDNA was not consistently prognostic on multivariable analysis: in Fang et al., it was significant univariately (HR = 3.28, 95% CI 1.18–9.13, *p* = 0.023) but not after adjustment (HR = 1.28, *p* = 0.67) [30], whereas Li et al. found a significant association with disease-free survival (DFS HR = 2.78, 95% CI 2.05–20.55, *p* < 0.05) [31]. In Gu et al., preoperative PTEN-specific ctDNA was the strongest independent predictor: positive patients had markedly shorter median DFS (4.01 vs. 33.27 months; HR = 7.53, 95% CI 3.08–18.42, *p* < 0.001) and overall survival (OS; 11.80 vs. 45.17 months; HR = 5.35, 95% CI 2.22–12.89, *p* < 0.001), both holding on multivariable analysis [32].

Postoperative ctDNA (MRD) was the most clinically informative timepoint across all four surgical cohorts. In Fang et al., MRD positivity independently predicted DFS (HR = 4.10, 95% CI 2.03–8.29, *p* < 0.001) and OS (HR = 5.38, 95% CI 2.65–10.95, *p* < 0.001), and a composite staging model adding ctDNA (TNMB, i.e., TNM supplemented by blood-based ctDNA) improved on standard TNM alone for DFS (C-index 0.80 vs. 0.65) and OS (0.77 vs. 0.69) [30]. Li et al. found postoperative MRD to be more accurate than preoperative ctDNA for recurrence prediction (sensitivity 83.3%, specificity 96.6%), and it remained independent on multivariable analysis (DFS HR = 303.75, *p* < 0.001); this extreme estimate, with a wide confidence interval, reflects the few postoperatively positive cases (*n* = 6 of 35) and should be read with caution [31]. Liu T. et al. confirmed independent prognostic value in the non-adjuvant subgroup, with a reduced median DFS (2.3 vs. not reached; multivariable HR = 184.6, *p* = 0.01) and OS (7.3 vs. not reached; HR = 25.8, *p* = 0.004), again with wide intervals from few positive cases (*n* = 4–6) [39]; notably, blood was collected at one week, a timepoint now recognized as susceptible to surgery-related cfDNA elevation, which the authors acknowledged [39]. Gu et al. showed that a gene-agnostic postoperative approach was not prognostic, but gene-specific tracking of TP53 and PIK3CA was: postoperative TP53 positivity predicted worse DFS (HR = 3.64, *p* = 0.005) and OS (HR = 3.29, *p* = 0.006) [32].

***Neoadjuvant and serial ctDNA cohorts***

Six studies tracked serial ctDNA dynamics in ESCC patients given neoadjuvant chemoradiotherapy (nCRT) or definitive chemoradiotherapy, with or without subsequent esophagectomy [33,34,35,36,37,38]. Baseline (pre-treatment) ctDNA carried no prognostic value: although detection was near-universal (99.2% in Yang Y. et al. [35]; 100% in Jiao et al. [33]), baseline status predicted neither pathological complete response (pCR) nor disease-free or overall survival in any of the six studies, which suggests that it reflects tumor burden rather than prognosis in the neoadjuvant setting.

On-treatment and post-treatment ctDNA, by contrast, were robust prognostic markers. Wang X. et al. found positivity at week 4 of chemoradiotherapy, which was the first independently prognostic timepoint (PFS HR = 3.35, 95% CI 1.10–10.22, *p* = 0.03), with hazard ratios rising at later timepoints (T2: HR = 5.45; T3: HR = 5.83) [38]; a rising trajectory from T1 to T2 was especially ominous (PFS HR = 9.09, 95% CI 1.40–59.27; OS HR = 10.25, 95% CI 1.56–67.15) [38]. Chen et al. found that post-treatment ctDNA at three months was prognostic for PFS (HR = 2.88, 95% CI 1.21–6.83, *p* = 0.012) and OS (HR = 3.67, 95% CI 1.41–9.55, *p* = 0.004) and that earlier clearance carried added value: ctDNA clearance before or during chemoradiotherapy gave clinical complete response rates of 100% and 83%, respectively, versus 50% and 33% for later or persistent positivity [36].

The landmark postoperative MRD timepoint was the single most informative ctDNA assessment in the neoadjuvant context. Jiao et al. showed a progressive escalation of hazard ratios across timepoints—pre-surgery (DFS HR = 4.29), landmark MRD within two months post-esophagectomy (DFS HR = 10.89, 95% CI 3.22–36.83, *p* < 0.001), and longitudinal MRD (DFS HR = 19.65, *p* < 0.0001)—all independent on multivariable analysis [33]. Notably, pathological complete response alone was no longer prognostic for DFS or OS once ctDNA was assessed, which indicates that ctDNA added prognostic information beyond pathological response in this study [33]; ctDNA-negative patients derived no benefit from adjuvant therapy, which suggests a role for ctDNA-guided de-escalation. Adding nivolumab to neoadjuvant chemotherapy increased the preoperative ctDNA-negativity rate versus placebo (89% vs. 62.5%, *p* = 0.02) [33].

Ko et al. reported the longest follow-up (≥5 years, ten serial timepoints): end-of-nCRT NFE2L2-specific ctDNA positivity predicted progression-free survival (univariate PFS HR = 4.36, 95% CI 1.44–13.23, *p* = 0.009; multivariable HR = 5.90, 95% CI 1.70–20.47, *p* = 0.005) and overall survival (univariate OS HR = 4.46, 95% CI 1.44–13.77, *p* = 0.009; multivariable HR = 4.75, 95% CI 1.40–16.15, *p* = 0.013), with a 158-day lead time before radiographic detection—the longest in the ESCC subgroup [34]; a composite model combining ctDNA with pathological N and T stage separated the highest- and lowest-risk groups by 8.5-fold in relapse risk [34]. Yang Y. et al., in the largest neoadjuvant ESCC series (*n* = 132), found that post-nCRT positivity identified a higher risk of distant metastases (28% vs. 5.3%) and of endoscopically negative clinical responses (19% vs. 2.7%) [35], and adding ctDNA to endoscopic biopsy halved the false-negative rate for residual disease (14.9% to 5.4%) [35]. Yue et al. showed that pre-surgical MRD achieved 100% sensitivity and 91% specificity for non-pCR, and postoperative MRD was associated with benefit from adjuvant immunotherapy in that study, a hypothesis-generating observation for MRD-guided treatment algorithms [37].

#### 3.3.2. Esophageal Adenocarcinoma (EAC)

Four studies examined ctDNA in patients with EAC, collectively enrolling 228 patients [40,41,42,43]. All four studies included patients receiving neoadjuvant therapy followed by curative esophagectomy. Key findings are summarized in Table 3b.

***Feasibility and methylation-based approaches***

Schoofs et al. reported the only methylation-based, tumor-agnostic cfDNA study in EAC, using cell-free reduced-representation bisulfite sequencing (cfRRBS) across seven longitudinal timepoints in 33 patients and 20 healthy donors [40]. This proof-of-concept work showed that a tumor tissue-independent approach is feasible: median baseline tumor fraction was higher in patients than in donors (2.2% vs. 1.6%, *p* = 0.032), and a tumor fraction above 15% was detectable in about two-thirds of patients sampled near clinical metastasis. Importantly, a significant tumor fraction spike occurred four to six days post-surgery (*p* = 0.002), attributable to surgical trauma-related cfDNA release rather than residual ctDNA—with implications for the timing of postoperative MRD assessment [40]. In high-tumor-fraction patients, copy-number profiling detected MYC, KRAS, EGFR, and NOTCH2 amplifications, which was confirmed in matched tissue in two of four cases. No survival analysis by ctDNA status was performed, consistent with the feasibility design.

***Tumor-agnostic versus tumor-informed platforms***

Two companion studies from the OCCAMS consortium directly compared tumor-agnostic and tumor-informed platforms in resected EAC [41,42]. The larger prospective cohort (*n* = 97) used the tumor-agnostic AVENIO 77-gene pan-cancer panel: postoperative ctDNA positivity—after mandatory clonal hematopoiesis (CHIP) correction, present in 23% of patients—independently predicted disease-free survival (DFS HR = 4.77, 95% CI 1.93–11.8, *p* < 0.001) and cancer-specific survival (CSS HR = 5.55, 95% CI 2.42–12.71, *p* = 0.0003) on multivariable analysis [41]. CHIP correction was critical: omitting it halved the hazard ratios (CSS HR 5.55 vs. 2.32), because TP53—the most common CHIP variant—is also the most common EAC driver [41]. Despite a high specificity (97%), sensitivity was only 35%, reflecting the inclusion of just 14 EAC-relevant genes among the 77 pan-cancer targets.

The companion retrospective study (*n* = 20) applied the tumor-informed Signatera platform—whole-genome sequencing of tumor tissue to define 16 patient-specific variants tracked by multiplex PCR-NGS—and doubled sensitivity versus the tumor-agnostic approach (80% vs. 35%), with 100% specificity and 100% positive predictive value [42]. All four postoperatively ctDNA-positive patients recurred, whereas none of the six preoperatively ctDNA-negative patients relapsed. The median lead time from ctDNA detection to clinical recurrence was 278 days (~9 months), exceeding 500 days in one patient [42]. A dynamic pattern—preoperative positivity converting to postoperative negativity—marked a favorable prognosis and tracked pathological response to chemotherapy.

***Post-chemoradiotherapy ctDNA and occult distant disease***

Hofste et al. conducted the largest prospective ctDNA study in locally advanced esophageal cancer at the time (*n* = 78, predominantly EAC), using a tumor-informed ultradeep hybrid-capture NGS panel of 15 genes across three serial timepoints [43]. Preoperative ctDNA at T0 (56.4% of patients) tracked tumor burden—gross tumor volume (*p* = 0.02), clinical N2 stage (*p* = 0.02), and ESCC rather than EAC histology (*p* = 0.03)—but was not independently prognostic, and early on-treatment ctDNA at T1 (around day 11) was similarly uninformative. By contrast, post-chemoradiotherapy preoperative ctDNA at T2, detected in only 10.3%, independently predicted disease progression (PFS HR = 2.8, 95% CI 1.1–6.8, *p* = 0.03) and death from any cause (OS HR = 2.9, 95% CI 1.2–7.1, *p* = 0.02) on multivariable analysis, controlling for pathological nodal status [43]. Crucially, T2 positivity occurred in patients achieving pathological complete response who later developed distant metastases—one with confirmed pCR and negative nodes developed liver metastases three months postoperatively—which suggests that post-chemoradiotherapy ctDNA can identify occult systemic disease missed by pathological response assessment [43].

#### 3.3.3. Gastric Cancer (GC)

Eight studies examined the role of ctDNA in patients with gastric cancer, collectively enrolling 357 patients [44,45,46,47,48,49,50,51]. All studies included patients undergoing curative-intent gastrectomy, with or without perioperative chemotherapy. Key findings are summarized in Table 3c.

***Preoperative ctDNA***

Preoperative ctDNA positivity rates varied markedly across gastric cancer studies, from 21% [51] to 80.4% [45], reflecting differences in panel size, detection threshold, and histological subtype mix. As with ESCC and EAC, preoperative ctDNA was not independently prognostic in most studies. Yuan et al. reported preoperative hazard ratios of 1.10 for recurrence-free survival and 1.13 for overall survival (both *p* = NS) [46]; Yang J. et al. found that preoperative positivity, though associated with disease stage (68% of stage III cases ctDNA-positive), lacked independent multivariable value [48]; and Kim et al. found preoperative status to be uncorrelated with recurrence (*p* = 0.637) [50]. Cabel et al. reported the lowest sensitivity in the cohort (21% at baseline), attributable to a small panel (39 genes with droplet digital PCR, ddPCR), absent ctDNA shedding in diffuse-type histology (0 of 6 positive at baseline), and complete ctDNA disappearance in all 18 evaluable patients after preoperative chemotherapy—even in those who later relapsed—which highlights the limitations of small-panel ddPCR [51].

***Postoperative MRD and serial ctDNA monitoring***

Postoperative ctDNA was the most clinically informative timepoint across all gastric cancer studies. Yang J. et al. found that postoperative positivity was associated with a 100% recurrence rate (7 of 7) versus 32% in negative patients (*p* = 0.0015), with DFS HR = 6.56 (*p* < 0.0001) and OS HR = 5.96 (*p* = 0.0007) [48]; longitudinal monitoring raised the signal further, any postoperative positivity yielding DFS HR = 14.78 (*p* < 0.0001) and OS HR = 7.66 (*p* = 0.002), with a 179-day (~six-month) median lead time before radiographic recurrence—among the longest reported [48]. Yuan et al., with the longest follow-up in this subgroup (52.2 months), showed escalating performance across timepoints: postoperative ctDNA HR for recurrence-free survival was 2.74, rising to 14.99 after adjuvant chemotherapy (post-ACT), with OS HRs of 2.53 and 11.88, respectively [46]; post-ACT ctDNA also gave the highest sensitivity (77.8%) and specificity (90.6%) [46]. Liu Z. et al. found that higher one-month postoperative ctDNA was associated with shorter overall survival (HR = 5.30) and higher recurrence risk (HR = 3.85), remaining an independent predictor of recurrence on multivariable analysis (HR = 4.00), and showed that a combined model adding serum markers (CEA, CA19-9, CA72-4) reached an area under the curve (AUC) of 0.940 for three-year survival—the strongest combined biomarker model in this literature [45]. The postoperative ctDNA sampling parameters of all included studies with postoperative assessment—including the interval from surgery to blood collection, its timing relative to adjuvant therapy, the number of serial postoperative timepoints, the number of evaluable postoperative samples, and whether ctDNA positivity preceded radiological recurrence—are summarized in Table 4.

***Peritoneal lavage ctDNA and alternative sampling strategies***

Bai et al. reported the only study using peritoneal lavage fluid (PLF) rather than plasma as the ctDNA source in 37 patients with stage III gastric cancer [44]. Preoperative PLF ctDNA positivity predicted peritoneal metastatic recurrence with 100% sensitivity but only 42% specificity (AUC = 0.86) and a preoperative HR of 4.82; combining PLF ctDNA with circulating tumor cells (CTCs) markedly improved performance (postoperative combined HR = 18.14, *p* < 0.05; AUC = 0.93) [44]. This targets a clinically important gastric recurrence pattern—peritoneal metastasis—that is poorly captured by blood-based ctDNA, owing to limited shedding into the systemic circulation from peritoneal implants. More fundamentally, peritoneal lavage and plasma sample distinct biological compartments and serve distinct clinical purposes: lavage fluid interrogates the local peritoneal cavity and is suited to predicting peritoneal (locoregional) metastasis, whereas plasma reflects systemic tumor-DNA shedding and is better suited to detecting hematogenous, distant recurrence. The two are, therefore, complementary rather than interchangeable, and their results are best interpreted separately rather than pooled—a separation we preserve by reporting this single lavage-based study apart from the plasma-based cohorts throughout this review.

***Methodological considerations: Clonal hematopoiesis and platform limitations***

Leal et al., in a prospective substudy of the CRITICS randomized trial (*n* = 50), provided a pivotal demonstration of the need for white-blood-cell DNA co-sequencing to correct for clonal hematopoiesis in gastric cancer [49]. Without WBC-based filtering, ctDNA failed to predict recurrence (*p* = 0.76); after correction, postoperative MRD positivity yielded a hazard ratio of 21.8 for both event-free and overall survival (95% CI 3.9–123.1, *p* < 0.001)—the highest in the gastric cancer subgroup—and all 11 ctDNA-negative patients remained alive and disease-free at a median of 42 months, with an 8.9-month lead time before clinical recurrence (the longest in the series) [49]. This study also found lower ctDNA shedding in diffuse-type gastric cancer—corroborated by Cabel et al. [51]—with implications for blood-based sensitivity in this subtype. Zhou et al., despite having the smallest series (*n* = 14), identified the CBLB mutation (15% of their cohort vs. <2% in The Cancer Genome Atlas) as a novel adverse prognostic biomarker, with a ~14-fold higher risk of death and progression (*p* < 0.01), though the small number of events warrants caution [47].

#### 3.3.4. Mixed Upper Gastrointestinal Tumors

Five studies examined ctDNA in mixed upper gastrointestinal cohorts—patients with esophageal cancer, gastroesophageal junction tumors, and gastric cancer—enrolling 525 patients [52,53,54,55,56]. These were the largest and most clinically heterogeneous series in this review, spanning curative perioperative settings and pathological complete response cohorts. Key findings are summarized in Table 3d.

***Perioperative MRD and postoperative surveillance***

Huffman et al. reported the largest real-world ctDNA series in upper gastrointestinal cancer to date—295 patients from more than 70 US institutions [56]. With the tumor-informed Signatera platform, postoperative MRD positivity independently predicted recurrence-free survival (HR = 10.7, 95% CI 4.3–29.3, *p* < 0.0001), with hazard ratios rising across surveillance: any postoperative positivity HR = 23.6, and surveillance-specific positivity HR = 17.7 (multivariable HR = 11.82, *p* < 0.001). Sensitivity was 85.7% at any postoperative timepoint and 80% during surveillance, with specificities of 95.5% and 98.3%, respectively [56]. Using a tumor-agnostic methylation-based ddPCR platform (TriMeth; C9orf50, KCNQ5, CLIP4) in 86 Danish patients, Iden et al. achieved comparable performance without tumor tissue: postoperative MRD positivity carried an RFS HR of 6.22 (*p* < 0.001) and an OS HR of 6.37 (*p* = 0.001; multivariable HR = 7.33) [54]. Early on-treatment ctDNA, after one cycle of perioperative chemotherapy, was also prognostic (RFS HR = 2.54, *p* = 0.005; OS HR = 2.23, *p* = 0.032), which suggests that ctDNA can provide early response information before imaging [54].

***ctDNA in pathological complete response cohorts***

Lander et al. addressed a clinically important question by enrolling only patients achieving pathological complete or near-complete response (TRG-0/1; *n* = 42) after neoadjuvant therapy across 11 US institutions [55]. Despite a pathological complete response, surveillance ctDNA positivity was associated with 100% recurrence (5 of 5), giving a surveillance RFS HR of 37.6 (95% CI 4.3–325.6, *p* < 0.001)—the highest RFS hazard ratio in the review—with a median 78-day lead time before radiographic recurrence; MRD-window positivity (within 16 weeks of surgery) was likewise associated with recurrence (HR = 6.2, *p* = 0.049) [55]. These findings suggest that pathological complete response may not be a definitive endpoint, showing that ctDNA can flag high relapse risk even in this favorable group.

***Neoadjuvant therapy monitoring and MRD-guided stratification***

In the prospective PLAGAST study (*n* = 62, mixed GEJ/gastric), Zaanan et al. showed a stepwise rise in prognostic performance across four serial ctDNA timepoints under FLOT-based neoadjuvant therapy [52]. During-neoadjuvant positivity carried an RFS HR of 6.17 (*p* = 0.002) and an OS HR of 4.71 (*p* = 0.022), post-neoadjuvant positivity showed an RFS HR of 5.26 and an OS HR of 7.35 (both *p* = 0.001), and postoperative MRD-window positivity showed an RFS HR of 12.94 (95% CI 4.23–39.59) and an OS HR of 14.54 (95% CI 4.54–46.6), both *p* < 0.0001 and independent on multivariable analysis [52]. All seven patients with post-neoadjuvant MRD positivity recurred (100%; 24-month RFS 0% vs. 62.8% in ctDNA-negative), and a combined model with pathological nodal involvement reached an RFS HR of 384.99 and concordance indices of 0.87 (RFS) and 0.91 (OS) [52]. ctDNA kinetics correlated with tumor regression grade (*p* = 0.035), with a 184-day lead time before radiographic recurrence—the longest in the mixed subgroup.

***Novel ctDNA kinetics definitions***

Hu et al., in a two-step observational study of 40 patients undergoing curative esophagectomy with or without neoadjuvant chemotherapy, introduced a novel kinetics-based definition of ctDNA positivity—a rise in tumor-informed mutation levels relative to pre-therapy baseline rather than an absolute threshold—using an in-house 250-gene panel [53]. By this definition, postoperative positivity independently predicted progression-free survival (HR = 19.1, 95% CI 2.21–164.85, *p* = 0.007), with 90.9% sensitivity and a 90-day median lead time before radiographic recurrence; four initially ctDNA-negative patients converted to positive during surveillance, which underscores the value of serial- over single-timepoint assessment [53].

## 4. Discussion

### 4.1. Principal Findings

This scoping review mapped ctDNA evidence for MRD detection and recurrence prediction across upper gastrointestinal cancers (27 studies, 1746 patients; ESCC, EAC, and gastric cancer). Its most reproducible signal—and the clearest clinically—was that postoperative ctDNA was more consistently prognostic than preoperative ctDNA. Preoperative positivity tracked tumor burden but seldom retained independent significance on multivariable analysis in ESCC [30,31], EAC [43], or gastric cancer [46,48,50,51]. Postoperative minimal residual disease behaved differently: assessed at a standardized landmark of roughly four to eight weeks, beyond the window of surgery-related cfDNA release, it was independently prognostic for disease-free and overall survival in every subgroup—ESCC [30,31,33], EAC [41,42,43], gastric cancer [45,46,48,49], and mixed cohorts [52,54,56]—with multivariable hazard ratios typically between 2.8 and 21.8. Extreme estimates from small ctDNA-positive subgroups (37.6 [55], 184.6 [39], and 384.99 [52]) carried very wide confidence intervals and are best set aside. The actionable timepoint is, therefore, postoperative, not preoperative.

A second pattern was that prognostic performance climbed across serial timepoints, consistent with ctDNA acting as a dynamic marker of active disease rather than a static measure of burden [33,38,46,52,56]—this is best illustrated by Jiao et al. (DFS HR rising from 4.29 pre-surgery to 10.89 landmark MRD and 19.65 longitudinal) [33] and Huffman et al. (MRD-window, anytime-postoperative, and surveillance HRs of 10.7, 23.6, and 17.7, respectively) [56]. Third, ctDNA detected recurrence ahead of imaging, with lead times of 78 [55] to 278 days [42], including 8.9 months in the gastric CRITICS substudy [49], 184 days in the mixed PLAGAST cohort [52], 179 days in a prospective gastric series [48], and 158 days after ESCC neoadjuvant chemoradiotherapy [34]. This defines a window for earlier intervention whose survival benefit remains to be proven prospectively. This interval has a lead-time advantage in the recognition of recurrence, which must be distinguished from a genuine improvement in patient outcomes: unless an effective intervention can be delivered at molecular relapse, earlier detection may merely advance the date at which recurrence is recognized without prolonging survival—the well-recognized problem of lead-time bias. The clinical value of early ctDNA detection is, therefore, contingent on the availability of effective ctDNA-directed interventions, which, as discussed in Section 4.3 and Section 4.5, have yet to be demonstrated in randomized trials.

Three further findings carry methodological weight. Tumor-informed platforms appeared to be more sensitive than tumor-agnostic ones, most directly in a single EAC cohort (80% vs. 35% at comparable specificity) [41,42]; because this rested largely on companion analyses of one consortium cohort rather than paired head-to-head testing, the difference should be interpreted cautiously—although methylation-based agnostic assays reached competitive specificity and prognostic performance (e.g., TriMeth, RFS HR 6.22) [54]. Correction for clonal hematopoiesis proved essential: in the CRITICS substudy, ctDNA was non-prognostic without white-blood-cell filtering but reached an MRD hazard ratio of 21.8 once corrected [49], and the same held in EAC [41]. Two histotype-specific limits emerged: diffuse-type gastric cancer sheds little ctDNA into plasma [49,51], and peritoneal recurrence is poorly captured in blood, with peritoneal lavage fluid outperforming plasma [44]. These reduced-detection scenarios most likely reflect genuine biological differences in ctDNA shedding rather than assay insensitivity alone: diffuse-type and signet-ring histologies combine low tumor cellularity with desmoplastic, stromal-rich growth that limits the release of tumor DNA into circulation, whereas peritoneal carcinomatosis sheds predominantly into the peritoneal cavity and has limited vascular access to the systemic compartment. Therefore, tumor biology and anatomical distribution—not only panel size or analytical sensitivity—contribute to false-negative plasma results, and distinguishing between these biological and technical sources is important for mechanistic interpretation: it calls for compartment-appropriate sampling, such as peritoneal lavage fluid, rather than for assay refinement alone.

These conclusions should be weighed according to the strength of the underlying evidence, not statistical significance alone. The included studies were generally small—a median of roughly 50 patients, ranging from 14 to 295—and several reported only a handful of recurrence events, which inflates hazard-ratio estimates and widens their confidence intervals (Section 4.4). Assay methodology also varied widely, from single-gene droplet digital PCR to tumor-informed multiplex PCR and broad sequencing panels, with detection thresholds spanning several orders of magnitude; because tumor-informed assays appear to be more sensitive than tumor-agnostic ones (on limited direct comparison), part of the between-study variation in detection and apparent prognostic strength may reflect methodology rather than biology. The risk of bias was likewise variable: on the Newcastle–Ottawa Scale, 14 of 27 studies were rated high quality, 12 moderate, and one low (Section 2.6), with the moderate-quality studies most often losing points for the absence of multivariable adjustment or for short follow-up. When these factors are weighed together, the strongest and most reproducible conclusion—the independent prognostic value of postoperative ctDNA—is supported by the convergence between the larger, multivariable-adjusted, higher-quality cohorts and the consistent direction of effect seen across the smaller studies, whereas more specific or single-study claims, such as particular predictive applications or head-to-head assay comparisons, rest on thinner evidence and warrant confirmation.

### 4.2. Comparison with Existing Literature

These findings align with and extend prior tumor-specific meta-analyses. In esophageal cancer, Zhang et al. (13 studies, 604 patients) reported pooled hazard ratios of 3.65 (OS), 6.08 (DFS/RFS), and 2.84 (PFS), with a stronger post-surgery OS HR of 9.02 [23]; Shen et al. confirmed that baseline ctDNA was non-prognostic, whereas post-neoadjuvant and post-surgery ctDNA predicted worse PFS and OS [24]; and Wang M. et al. (22 studies) showed escalating pooled PFS hazard ratios from baseline (1.64) through to post-neoadjuvant (3.97) and surveillance (5.42) [57]. In gastric cancer, Mi et al. reported pooled recurrence relative risks of 1.79 (preoperative) and 3.17 (postoperative) and hazard ratios of 6.37 (RFS) and 4.58 (OS) [25]; later individual studies—Leal et al. (HR 21.8 after CHIP correction) [49] and Yang J. et al. (longitudinal DFS HR 14.78) [48]—substantially exceed these pooled estimates, which indicates that earlier, weaker studies attenuate pooled effects.

Our findings fit the broader liquid-biopsy literature [58] and available evidence grading. Allan et al. gave ctDNA a high GRADE rating for MRD detection in localized esophagogastric cancer and a moderate rating for surveillance, stressing CHIP correction, collection timing, and tumor-informed sensitivity [59], while Lee et al. noted greater ctDNA shedding in ESCC than in adenocarcinoma and added value in HER2-amplified disease [60]. Epigenetic methods are promising: Zhao et al. reported methylation-based early detection (76.2% sensitivity, 86.3% specificity) [61]; broader multi-cancer methylation detection has been shown [62]; and, in the MRD setting, Schoofs et al. (cfDNA methylation in EAC) [40] and Iden et al. (TriMeth) [54] suggest that methylation could close the gap with tumor-informed mutation-based assays. What sets this review apart is its scope: covering ESCC, EAC, and gastric cancer in one framework and including studies up to April 2026, it shows that postoperative ctDNA was more prognostic than preoperative ctDNA as a consistent pattern across histology, treatment, and geography. Even so, the included cohorts differed substantially in disease stage, the use of neoadjuvant therapy, surgical approach, assay methodology, the timing of blood collection, and definitions of recurrence, and the ESCC and EAC studies derived predominantly from Asian and Western populations, respectively; this consistency is, therefore, best interpreted as a highly reproducible association across diverse settings rather than a universal biological principle, and it requires prospective validation using standardized sampling and reporting protocols.

The decision to consider esophageal squamous cell carcinoma, esophageal adenocarcinoma, and gastric cancer together warrants explicit justification. These entities were grouped not because they are biologically equivalent—they differ materially in their molecular drivers, standard treatment, patterns of relapse, and ctDNA shedding—but because they share an anatomical region, a curative-intent paradigm of neoadjuvant or perioperative therapy followed by resection, and comparable postoperative surveillance, and because no previous synthesis had mapped ctDNA MRD evidence across all three within a single framework. A scoping design suits this aim, identifying principles that recur across the group while surfacing the histotype-specific detail that single-tumor reviews miss. The conclusions of this review accordingly differ in their generalizability. The cross-cutting findings—that postoperative MRD is more informative than preoperative ctDNA and that prognostic performance rises with serial sampling—were reproduced across all three cancers and are reasonably transferable, as is the tumor-agnostic requirement for clonal hematopoiesis correction, demonstrated here in the esophageal adenocarcinoma and gastric cohorts. Others are disease-specific and should not be generalized: the low plasma yield of diffuse-type and signet-ring gastric cancer, the need for peritoneal-compartment sampling to capture gastric peritoneal recurrence, the differences in ctDNA shedding between esophageal squamous cell carcinoma and adenocarcinoma, and the predominantly Asian (ESCC) versus Western (EAC) provenance of the evidence all limit cross-histology extrapolation and should be respected when translating these conclusions into tumor-specific protocols and trials.

### 4.3. Clinical Implications

The most immediate use is postoperative risk stratification. MRD-positive patients had recurrence rates approaching 100% [48,55,56] and shorter survival, independent of stage, nodal status, and tumor regression grade. Notably, in individual studies, ctDNA provided independent prognostic information beyond pathological complete response [33,55], which suggests that pCR alone may be an imperfect endpoint. Even so, ctDNA is best understood as adding to, rather than replacing, conventional staging and pathological response assessment—refining established risk stratification rather than superseding it. This supports two complementary strategies—de-escalation in ctDNA-negative patients, in whom adjuvant therapy yielded no demonstrable benefit [33,37], with DYNAMIC providing proof of principle in colon cancer [22], and escalation or early salvage in MRD-positive patients within the 78–278-day lead-time window. Serial monitoring could refine surveillance: with specificities of 95–98% in the largest series [54,56], ctDNA-triggered imaging—analogous to IMPROVE-IT2 in colorectal cancer [63]—could reduce unnecessary scans while accelerating recurrence detection. These applications should be framed by biomarker function: the included studies establish postoperative ctDNA predominantly as a prognostic marker that stratifies the risk of recurrence, whereas its use as a predictive biomarker—one that identifies who will benefit from a specific treatment and thereby guides escalation or de-escalation—is not established by these largely observational data, in which treatment was not randomized by ctDNA status; randomized, biomarker-stratified trials are required (Section 4.5). The de-escalation and escalation strategies outlined above should accordingly be regarded as hypotheses to be tested rather than as evidence-based treatment recommendations.

Platform choice should depend on context. Tumor-informed assays offer greater sensitivity [41,42] (an advantage whose magnitude depends on assay design and analytical methodology, such as panel breadth and variant-calling thresholds) but require tumor tissue and longer turnaround, whereas tumor-agnostic and methylation-based assays can be used in any patient; clonal hematopoiesis correction is broadly advisable, but its necessity and optimal implementation likewise depend on assay design and analytical methodology—the risk of misattributing clonal hematopoiesis variants to tumors is greatest for tumor-agnostic, broad-panel assays and is mitigated by paired white-blood-cell sequencing, whereas tumor-informed assays anchored to pre-specified tumor-specific variants are inherently less susceptible [41,49], and consensus guidance on ctDNA assay use is now available [64,65]. These differences also translate into practical trade-offs for clinical adoption: tumor-informed assays additionally require individualized, patient-specific panel design and sufficient tumor tissue for baseline sequencing, whereas tumor-agnostic methylation-based assays use a fixed, tissue-independent panel that is simpler to standardize and potentially more scalable and cost-effective, generally at the price of lower sensitivity. The optimal platform in a given setting is, therefore, likely to depend on tissue availability, acceptable turnaround, and local resources as much as on analytical sensitivity alone.

The possibility of false-negative results warrants balanced emphasis. Whereas a positive postoperative ctDNA result is a strong and consistent predictor of recurrence, a negative result carries more limited negative predictive value and should not be equated with the absence of residual disease. False negativity is expected wherever tumor-DNA shedding into plasma is low—most notably in diffuse-type and signet-ring gastric cancer, which combine low tumor cellularity with a desmoplastic, stromal-rich growth pattern, and in peritoneum-confined disease with limited vascular access to the systemic circulation. Lower diffuse-type shedding was reported directly in the included evidence [49] and corroborated by a cohort in which no diffuse-type tumor was ctDNA-positive at baseline (0 of 6) [51]. Very-low-burden or early minimal residual disease lying below the assay’s limit of detection is a further, histology-independent source of false negativity. In these low-shedding settings, a negative plasma ctDNA result should, therefore, be interpreted cautiously and should not, by itself, be used to withhold standard adjuvant treatment or to relax surveillance; compartment-appropriate sampling—such as peritoneal lavage fluid [44]—or complementary modalities may be needed to avoid missing residual disease.

These implications should be read within the established biomarker development pathway, which separates analytical validity (the accuracy and reproducibility with which an assay detects and quantifies ctDNA) from clinical validity (the strength and consistency of the association between ctDNA status and the clinical outcome) and from clinical utility (the demonstration that acting on the biomarker improves patient outcomes) [65]. Judged against this framework, the evidence mapped here offers substantial support for clinical validity, as postoperative ctDNA was independently and reproducibly associated with recurrence across histologies, treatments, and geographic settings, and it bears directly on analytical validity, given the decisive influence of tumor-informed assay design and mandatory clonal hematopoiesis correction on performance. Clinical utility, by contrast, remains unproven: no randomized trial has yet shown that ctDNA-guided treatment decisions improve survival or other patient-important outcomes in upper gastrointestinal cancer. The strong and consistent prognostic associations documented here, therefore, support postoperative ctDNA as a clinically valid risk marker and a rational basis for interventional trials rather than as an intervention with proven clinical benefit.

### 4.4. Limitations

Several limitations temper these conclusions. As a scoping review, this study was not designed to pool data, and no summary effect estimates were calculated; readers seeking such estimates are referred to the meta-analyses discussed above. The approach suits a heterogeneous, fast-moving field and avoids a false sense of precision, but it rules out formal statistical comparison between tumor types, platforms, or clinical settings. A further consequence of the scoping design concerns the potential overlap between cohorts: a small number of included studies derive from shared parent datasets—the two OCCAMS esophageal adenocarcinoma reports, which are companion analyses of the same consortium cohort, and the two real-world Signatera esophagogastric studies, which draw on the same commercial testing program and share several investigators—so a degree of patient overlap between these paired reports cannot be excluded. Because no data were pooled and no summary effect estimate was calculated, such overlap does not bias any aggregate statistic; nonetheless, the total of 1746 patients should be read as the sum of the cohorts reported by the included studies rather than as a count of unique individuals, and each pair of reports is best interpreted as complementary analyses rather than independent replications.

The underlying evidence is highly heterogeneous: detection platforms ranged from single-gene droplet digital PCR to tumor-informed multiplex PCR, gene panels from 5 to 1021 targets, and variant allele fraction thresholds from 0.001% to 5%, with widely varying blood-collection timing and follow-up. Most studies were small, single-center, retrospective cohorts (median ~50 patients), which limits the power to detect independent associations and widens imprecision—hence the occasionally extreme confidence intervals (e.g., HR 184.6 [39]). Prospective, multi-center studies with pre-specified sample sizes are needed for reliable, generalizable estimates. For the same reasons, the absolute magnitude of individual hazard ratios should not be compared directly across studies: these estimates derive from different patient populations, assay platforms, statistical models, follow-up durations, and outcome definitions, and their wide, frequently overlapping confidence intervals preclude meaningful ranking. The more informative conclusion is the consistent direction and strength of the association between postoperative ctDNA and recurrence across studies, rather than differences in absolute hazard-ratio magnitude. Relatedly, the independent prognostic value of postoperative ctDNA was established predominantly through statistical significance on multivariable analysis, adjusting for stage and other covariates, whereas formal comparisons of discriminative performance against established staging—for example, by concordance index or area under the curve—were reported in only a few studies (such as the composite TNMB model of Fang et al., C-index 0.80 versus 0.65). Postoperative ctDNA is, therefore, best regarded as providing independent, additive prognostic information rather than as having demonstrated superiority over validated staging systems, consistent with the cautious wording adopted throughout the manuscript.

A further source of variability lies in sample completeness and pre-analytical handling, which were inconsistently reported across the included studies. Few studies documented the rate of assay failures or how many enrolled patients lacked an evaluable ctDNA sample at each timepoint. Examples include samples excluded for insufficient cell-free DNA yield or for failing sequencing quality control; where such patients are dropped from analysis and differ systematically from those retained, incomplete or non-random sampling can introduce selection bias and inflate apparent performance. Pre-analytical factors known to affect cell-free DNA integrity and tumor-fraction recovery—the blood-collection tube used (standard EDTA versus cell-stabilizing tubes), the interval and temperature between venipuncture and plasma separation, the centrifugation protocol, and plasma storage conditions—were likewise seldom specified, yet differences in these steps can materially alter ctDNA detection and contribute to the between-study heterogeneity noted above. Standardized, transparently reported pre-analytical protocols, together with complete accountings of missing and failed samples, would reduce these sources of bias and improve comparability across studies.

Variability in ctDNA detection also has a biological basis that extends beyond residual tumor burden. The amount of tumor DNA released into circulation is modulated by several factors—including tumor vascularity, proliferation and cellular turnover rate, the extent of necrosis, the metastatic pattern, and anatomical location [14]—that differ between tumors, between histological subtypes, and over the course of disease. Because ctDNA is, thus, only an indirect surrogate of tumor burden, these determinants offer a more comprehensive explanation for the differences in detection observed among studies and across tumor types—the low plasma yield of diffuse-type gastric cancer and of peritoneal disease being specific examples—and warrant consideration when ctDNA performance is compared across settings.

Generalizability is further limited by representation and follow-up. The literature is geographically skewed—every ESCC study, and 15 of 27 overall, came from China—which limits extrapolation, while the EAC data come mainly from Western European centers. Follow-up is mostly short, with only one study beyond five years [34], so the long-term value of MRD may be underestimated. Finally, clonal hematopoiesis correction was applied inconsistently, which can weaken or abolish the ctDNA signal [41,49]; publication bias cannot be excluded; and studies appearing after the April 2026 search are not captured. The literature search itself has limitations that bear on reproducibility: we searched PubMed, Scopus, and the Cochrane Library but not Embase or grey-literature sources, so a small number of eligible reports may have been missed; consistent with the scoping framework, the review was not registered in a public registry and followed no a priori published protocol (Section 2.1); retrieved records were deduplicated within a reference-management system, although the number removed at this initial deduplication step was not separately reported (a further six duplicate reports were removed at full-text assessment); and supplementary citation searching was confined to backward hand-searching of the reference lists of included and related studies (which yielded 14 additional reports) rather than systematic forward citation tracking.

A final caveat concerns the interpretation of the reported lead times. In most of the included studies, recurrence was ascertained by imaging-based surveillance whose frequency was generally neither standardized across ctDNA strata nor documented in relation to ctDNA status, and blinding of the treating team to ctDNA results was seldom reported. Where a positive ctDNA result could prompt additional or earlier imaging, recurrence would be verified more intensively in ctDNA-positive than in ctDNA-negative patients—a form of verification (work-up) bias—so that the reported intervals by which ctDNA positivity preceded radiological recurrence may partly reflect differences in surveillance intensity rather than solely earlier biological detection. The net direction of this effect is uncertain: more frequent imaging of ctDNA-positive patients would, if anything, tend to shorten rather than lengthen the measured lead time, so the concern is one of confounding by non-standardized surveillance rather than systematic overestimation. This possibility is attenuated in the cohorts for which ctDNA was analyzed retrospectively on banked samples, in which the results could not have guided imaging, but it cannot be excluded and is difficult to quantify retrospectively. The reported lead times, therefore, warrant cautious interpretation, and prospective designs with predefined, ctDNA-blinded imaging schedules would be needed to disentangle surveillance intensity from biological detection. Beyond this within-study concern, the reported lead-time values should not be compared directly across studies. Sampling frequency ranged from a single postoperative sample to repeated serial monitoring, imaging-surveillance schedules and recurrence definitions differed, and the studies varied in their design, tumor type, and treatment; a longer reported lead time in one study, therefore, need not reflect earlier biological detection than a shorter one in another. Mirroring the caution applied above to hazard-ratio magnitudes, the reproducible finding that ctDNA can precede radiological recurrence is more secure than findings regarding any specific interval, and differences in reported lead times across studies should be regarded as at least partly methodological.

### 4.5. Future Directions

Three priorities stand out: assay standardization, interventional evidence, and biomarker integration. Consensus reporting standards—along the lines of the NCI Colon and Rectal–Anal Task Forces white paper on colorectal cancer [66]—should set minimum analytical requirements, mandatory CHIP correction, and harmonized timing and positivity thresholds. Whether current evidence supports a single preferred sampling window merits explicit comment. The postoperative landmark most consistently associated with independent prognostic value lies at roughly four to eight weeks after surgery—late enough for surgery-related cell-free DNA to clear, yet early enough to inform adjuvant decisions—and this window was adopted by several of the strongest studies. As Table 4 shows, however, the interval from surgery to the first postoperative sample ranged from under one week to sixteen weeks across these studies, and no study directly compared candidate landmarks. The available data, therefore, support a four-to-eight-week window as a reasonable default rather than defining a definitive optimal landmark; prospectively comparing sampling timepoints should be an explicit objective of the standardization efforts advocated above. Crucially, no randomized trial has yet shown that ctDNA-guided management improves survival in upper gastrointestinal cancers. De-escalation trials in ctDNA-negative patients (modeled on DYNAMIC [22]), MRD-triggered intervention trials, and treatment-escalation trials are all needed, and the PLAGAST design [52] and an embedded nivolumab MRD substudy [33] offer early frameworks.

Histotype-specific validation is required, for example, alternative sampling (peritoneal lavage fluid, circulating tumor cells) in diffuse-type and signet-ring disease and larger, geographically diverse EAC cohorts to confirm the OCCAMS findings [41,42]. Combining methylation- and mutation-based assays [40,54] and pairing ctDNA with circulating tumor cells, cell-free RNA, and tumor-educated platelets in a multimodal liquid biopsy [58,67,68] may improve sensitivity in low-shedding tumors, and complementary functional imaging such as 18F-FDG PET-CT deserves prospective study [36]. Finally, because prognostic information resides in ctDNA kinetics rather than single-timepoint positivity, machine-learning models trained on serial trajectories—supported by large consortium datasets [69]—represent a promising direction.

Framed formally, these priorities map onto the biomarker qualification pathway set out in Section 4.3 [65]: analytical validation of standardized assays, clinical validation in prospective multi-center cohorts, and—the decisive current gap—demonstration of clinical utility in randomized interventional trials. Advancing ctDNA through these sequential stages, ideally within recognized regulatory biomarker-qualification frameworks, will be a prerequisite for regulatory endorsement and for the incorporation of ctDNA-guided strategies into treatment guidelines and routine clinical practice.

## 5. Conclusions

This review offers the most comprehensive mapping to date of ctDNA for minimal residual disease detection and recurrence prediction across esophageal squamous cell carcinoma, esophageal adenocarcinoma, and gastric cancer. In all three, postoperative ctDNA emerges as a consistent, independent prognostic biomarker: it is more informative than preoperative measurement, grows stronger with serial monitoring, and often anticipates radiological recurrence—while a pathological complete response may be an imperfect surrogate in its presence. ctDNA is, thus, best positioned as a complement to established measures such as staging and pathological response rather than as a replacement for them. Tumor-informed assays showed the highest sensitivity in the limited direct comparisons available, methylation-based agnostic platforms are a practical fallback when tumor tissue is unavailable, and correction for clonal hematopoiesis is indispensable for tumor-agnostic assays; blood-based testing still falls short in diffuse-type and peritoneal disease.

These conclusions should be read against the limitations of the current evidence, which remains heterogeneous and largely observational: the included studies varied widely in their design, ctDNA platform, sampling timepoint, and follow-up; most were small and single-center; comparatively few adjusted for established prognostic factors; and none tested whether acting on a ctDNA result changes patient outcomes. Postoperative ctDNA is, therefore, best regarded, at present, as a promising prognostic biomarker rather than as a validated, clinically actionable one. The decisive step now is to move from observational to interventional evidence. Adequately powered randomized trials of ctDNA-guided de-escalation in ctDNA-negative patients and MRD-triggered intervention in MRD-positive patients, supported by international standardization of assay methodology and reporting, will determine whether ctDNA can progress from a prognostic marker to a tool that truly reshapes the postoperative care of patients with upper gastrointestinal cancer. In summary, postoperative ctDNA currently represents a clinically valid prognostic biomarker whose clinical utility—the demonstration that acting on a ctDNA result improves patient outcomes—awaits confirmation in prospective, interventional trials.

## Figures and Tables

**Table 2:** (**a**) Characteristics of included studies—esophageal squamous cell carcinoma (ESCC). (**b**) Characteristics of included studies—esophageal adenocarcinoma (EAC). (**c**) Characteristics of included studies—gastric cancer (GC). (**d**) Characteristics of included studies—mixed upper GI tumors (esophagus/GEJ/stomach).

(**a**)							
**Author/Year**	**Country**	**Study Design**	**N**	**M/F**	**Median Age**	**Median FUP (m)**	**Stage**
Fang C.Y./2025 [30]	P.R. China	Retrospective	125	95/30	63	40.0	II: 68; III: 48; IVA: 9
Jimin Li/2025 [31]	P.R. China	Prospective	35	18/17	57	24	I: 6; II: 19; III: 10
Rentong Gu/2025 [32]	P.R. China	Prospective	54	44/10	65	36	I: 14; II: 16; III: 22; IVA: 2
Heng Jiao/2025 [33]	P.R. China	Prospective RCT	65	NR	NR	24.9	NR
Ko J.M.Y./2025 [34]	P.R. China	Prospective	52	NR	NR	≥60	NR
Yang Yang/2025 [35]	P.R. China	Prospective	132	111/21	65	12	NR
Baoqing Chen/2024 [36]	P.R. China	Prospective	42	32/10	NR	27.6	I-III: 62% (26/42); IVA: 38% (16/42)
Pinli Yue/2024 [37]	P.R. China	Prospective RCT	38	32/6	62	17	II: 10; III: 27; IVA: 1
Xin Wang/2022 [38]	P.R. China	Prospective	40	34/6	64	20.6	II: 2; III: 23; IVA: 6; IVB: 9
Liu T./2021 [39]	P.R. China	Retrospective	53	44/9	65	34.8	I: 12; II: 22; III: 19
(**b**)							
**Author/Year**	**Country**	**Study Design**	**N**	**M/F**	**Median Age**	**Median FUP (m)**	**Stage**
Schoofs K./2024 [40]	Belgium	Prospective observational	33	28/5	NR	NR	NR
Ococks E./2021 [41] (Ann Oncol)	UK	Prospective national cohort	97	83/14	68.2	32.9	All cT3/T4
Ococks E./2021 [42] (Gastroenterology)	UK	Retrospective (OCCAMS subset)	20	17/3	62.8	Up to 75+	I: 1; II: 5; III: 12
Hofste L.S.M./2022 [43]	Netherlands	Prospective observational	78	60/18	67	PFS 28; OS 30	IB: 6; IIA: 20; IIB: 7; IIIA: 29; IIIB: 16
(**c**)							
**Author/Year**	**Country**	**Study Design**	**N**	**M/F**	**Median Age**	**Median FUP (m)**	**Stage**
Bai L./2025 [44]	P.R. China	Prospective	37	26/11	68.6	14.6	All stage III
Liu Z./2025 [45]	P.R. China	Prospective	59	41/18	≥60:47	24.9	II: 25; III: 33; IV: 1
Yuan S.Q./2023 [46]	P.R. China	Prospective	100	68/32	NR	52.2	II: 37; III: 63
Zhou H./2023 [47]	P.R. China	Prospective	14	13/1	NR	NR	III: 6; IV: 8
Yang J./2020 [48]	P.R. China	Prospective	46	38/8	54	29.1	0: 2; I: 9; II: 12; III: 23
Leal A./2020 [49]	Netherlands/Sweden/Denmark	Prospective (CRITICS RCT substudy)	50	NR	NR	42	I: 10; II: 15; III: 13; IV: 2
Kim Y.W./2019 [50]	Korea	Retrospective	19	17/2	60	12	II: 4; III: 14; IV: 1
Cabel L./2019 [51]	France	Prospective proof-of-concept	32	24/8	65	26	NR
(**d**)							
**Author/Year**	**Country**	**Study Design**	**N**	**M/F**	**Median Age**	**Median FUP (m)**	**Stage**
Zaanan A./2025 [52]	France	Prospective	62	39/23	66	29	0: 3; I: 16; II: 26; III: 17
Hu Q./2025 [53]	Japan	Two-step observational (retrospective pilot *n* = 6 + prospective *n* = 34)	40	32/8	67	C1: 22.6; C2: 13.9	I: 11; II: 13; III: 13; IV: 3
Iden C.R./2025 [54]	Denmark	Prospective	86	72/14	65.2	26.7	NR
Lander E.M./2024 [55]	USA	Retrospective real-world multi-center	42	33/9	NR	28.5	I: 3; II: 10; III: 28; IV: 1
Huffman B.M./2022 [56]	USA	Retrospective real-world multi-center	295	196/99	NR	13.9	I: 29; II: 64; III: 119; IV: 83

FUP: follow-up; M/F: male/female; NR: not reported; RCT: randomized controlled trial. Clinical stage reported according to AJCC/UICC staging at time of study enrollment. Stage 0 not shown where *n* = 0 for all studies. For Jiao 2025 [33], *N* = 65 refers to the ctDNA-evaluable subgroup; demographic and staging data for this subgroup are not separately reported in the original publication (full ITT cohort *N* = 90). OCCAMS: Esophageal Cancer Clinical and Molecular Stratification consortium; EMR: endoscopic mucosal resection. Hofste stage distribution refers to AJCC clinical T/N stage at enrollment (locally advanced cohort, cT2–cT4). Ococks (Gastroenterology) stages refer to pathological T stages; the cohort of *N* = 20 comprises 17 patients treated by esophagectomy and 3 by EMR, and pathological T-stage data are available for the 18 patients with surgical specimens. CRITICS: ChemoRadiotherapy after Induction chemotherapy In Cancer of the Stomach trial. For Leal et al., *N* = 50 reflects the total enrolled cohort; pathological staging was available for 40 patients who underwent curative-intent surgery (7 patients did not proceed to surgery, and 3 had missing staging data). GEJ: gastroesophageal junction; C1/C2: Cohort 1/Cohort 2 (Hu Q.).

**Table 3:** (**a**) Role of ctDNA in recurrence detection and outcome prediction in esophageal squamous cell carcinoma (ESCC)—Surgical cohorts. (**b**) Role of ctDNA in recurrence detection and outcome prediction in esophageal adenocarcinoma (EAC). (**c**) Role of ctDNA in recurrence detection and outcome prediction in gastric cancer (GC). (**d**) Role of ctDNA in recurrence detection and outcome prediction in mixed upper gastrointestinal tumors.

(**a**)								
**Study/Year**	**N**	**Setting & Design**	**ctDNA Method**	**Key Timepoint & Positivity Rate**	**Sensitivity/Specificity, PPV/NPV**	**DFS/RFS HR (95% CI), *p***	**OS HR (95% CI), *p***	**Key Finding/Lead Time**
Upfront surgical cohorts								
Fang C.Y. 2025 [30] China	125	Retrospective; curative esophagectomy; FUP 40.0 m; recurrence 43.2%	Panel NGS (multi-gene); tumor-informed; plasma	Preop: 79.6% positive (86/108); postop MRD (7–14 d): 48.0% positive (60/125); clearance: 45.4% (39/86)	Postop MRD: Sens 83.3%, Spec 60.4% (1 yr)	Preop (MV): NS (HR = 1.28, *p* = 0.67); postop MRD+ (independent): HR = 4.10 (95% CI 2.03–8.29), *p* < 0.001; non-clearance: HR = 3.55 (95% CI 1.95–6.47), *p* < 0.001	Postop MRD+ (independent): HR = 5.38 (95% CI 2.65–10.95), *p* < 0.001	Postop ctDNA is the key biomarker (independent on MV). TNMB staging improved on TNM alone (C-index DFS 0.80 vs. 0.65; OS 0.77 vs. 0.69).
Jimin Li 2025 [31] China	35	Prospective; curative surgery; 2 yr FUP; MRD monitoring	NGS (method NR); tumor-informed; plasma	Preop: 54.3% positive (19/35); postop ~1 m MRD: 17.1% positive (6/35)	Preop: Sens 100%, Spec 55.2%, PPV 31.6%; postop MRD: Sens 83.3%, Spec 96.6%	Preop+: HR = 2.78 (95% CI 2.05–20.55), *p* < 0.05; postop MRD+ (independent): HR = 303.75 (95% CI not reported; unstable estimate, small N), *p* < 0.001	NR	Postop MRD is an independent predictor on MV. MRD+ is linked to immune escape. Wide CI reflects small N.
Rentong Gu 2025 [32] China	54	Prospective; curative surgery; FUP 36 m (median); recurrence 64.8%	Panel NGS; gene-specific ctDNA (PTEN, TP53, PIK3CA); tumor-informed; plasma	Preop: 98.1% positive (53/54); postop MRD: 22.2% positive (TP53 11.1%, PIK3CA 11.1%); clearance: 55.6%	Not formally calculated	Preop PTEN+ (independent): DFS HR = 7.53 (95% CI 3.08–18.42), *p* < 0.001; postop TP53+: DFS HR = 3.64 (95% CI 1.48–8.97), *p* = 0.005	Preop PTEN+ (independent): OS HR = 5.35 (95% CI 2.22–12.89), *p* < 0.001; postop TP53+: OS HR = 3.29 (95% CI 1.34–8.06), *p* = 0.006	Gene-specific tracking essential: the agnostic postop approach is not significant. PTEN ctDNA+ is the strongest independent predictor.
Liu T. 2021 [39] China	53	Retrospective; curative esophagectomy (no neoadjuvant); FUP 34.8 m; stages 0–III; primary analysis non-adjuvant group (*n* = 23)	61-gene capture NGS; tumor-informed + tumor-agnostic (parallel); paired WBC sequencing; plasma	Pre-surgery cfDNA: 38/53 evaluable (73.7% concordance with FFPE); postop (1 week): 15.8% positive (6/38)	Tumor-informed: Sens 60%, Spec 95.45%; tumor-agnostic: Sens 80%, Spec 63.64%	Non-adjuvant group (independent on MV): DFS HR = 184.6 (95% CI 3.6–9576.9), *p* = 0.01 (wide CI; *n* = 4 ctDNA+); median DFS ctDNA+: 2.3 m vs. NR	Non-adjuvant group (independent on MV): OS HR = 25.8 (95% CI 2.7–242.6), *p* = 0.004; median OS ctDNA+: 7.3 m vs. NR	Blood collected at 1 week postop (within surgical-trauma window; key limitation). First ESCC study (>50 patients) with paired pre- and post-surgical cfDNA. Lead time is not reported.
Neoadjuvant and serial ctDNA cohorts								
Heng Jiao 2025 [33] China	65	Prospective RCT (nivolumab + chemo vs. placebo, then MIE); landmark MRD design; FUP 24.9 m	Panel NGS; tumor-informed; serial (4 timepoints); plasma	T0 baseline: 100% positive (not prognostic) T2 landmark (≤2 m post-MIE): 25.6% positive (11/43); longitudinal MRD+: 10 relapsed vs. 1/30 MRD−	T2 landmark MRD: PPV 0.73, NPV 0.88 (superior to preop: PPV 0.62, NPV 0.80)	T1 pre-MIE: HR = 4.29 (95% CI 1.68–10.93), *p* < 0.001; T2 landmark: HR = 10.89 (95% CI 3.22–36.83), *p* < 0.001; longitudinal MRD+: HR = 19.65 (95% CI 4.26–90.58), *p* < 0.0001	T2 landmark: *p* < 0.001; longitudinal MRD+: *p* = 0.003; MRD-: no difference with vs. without adjuvant	pCR no longer prognostic once ctDNA assessed; MRD- patients can safely omit adjuvant therapy. Nivolumab increased preoperative MRD negativity (89% vs. 62.5%, *p* = 0.02).
Ko J.M.Y. 2025 [34] China	52	Prospective; nCRT, then surgery; ≥5 yr FUP (longest); 10 serial timepoints	AVENIO tumor-informed; NFE2L2-specific (assay Sens 98%, Spec 98.6%); plasma	T0 baseline: 18.2% positive (not prognostic); end of nCRT: 8.3% positive; pre-surgery: 16.7% positive; post-surgery: 12.8% positive; recurrent (≥2 timepoints): 9.8% positive	End-of-nCRT NFE2L2+: independent prognosticator on MV	End of nCRT+: UV PFS HR = 4.36 (95% CI 1.44–13.23), *p* = 0.009; MV PFS HR = 5.90 (95% CI 1.70–20.47), *p* = 0.005; pre-surgery+: HR = 2.46 (95% CI 1.11–5.47), *p* = 0.028; post-surgery+: HR = 3.66 (95% CI 1.45–9.26), *p* = 0.006; recurrent+: HR = 3.61 (95% CI 1.34–9.71), *p* = 0.011	End of nCRT+: OS HR = 4.75 (95% CI 1.40–16.15), *p* = 0.013	Only study with ≥5 yr FUP and 10 timepoints. Risk model (ctDNA + pN + pT): 8.5-fold relapse-risk difference. Lead time 158 days (longest time in ESCC subgroup).
Yang Yang 2025 [35] China	132	Prospective; nCRT, then surgery; minimum 12 m FUP (not powered for survival)	Tumor-informed NGS panel; serial sampling; plasma	Baseline: 99.2% positive (near-universal); post-nCRT (CRE-1)+: 56.8% (75/132)	Post-nCRT ctDNA+: distant metastases 28% vs. 5.3%; biopsy false-negative rate reduced 14.9% to 5.4%	Formal HR NR (not powered for survival)	NR	Highest baseline ctDNA rate in review (99.2%). ctDNA detects systemic recurrence beyond endoscopy; adding ctDNA to biopsy reduces false-negative rate to 5.4%.
Baoqing Chen 2024 [36] China	42	Prospective; definitive CRT + toripalimab (PD-1); FUP 27.6 m	Panel NGS (cfDNA); top mutations TP53 68%, CDKN2A 20%, NFE2L2 15%; plasma	T0: 73% positive (29/40; not prognostic); T1 (wk 3 CRT): 44% positive; T2 (3 m post-CRT): 27% positive	T1 for cCR: Sens 70%, Spec 82%; T2 for cCR: Sens 87%, Spec 64%; PET-CT: Sens 83%, Spec 100%	T0: NS (HR = 1.50); T1 (wk 3): PFS HR = 2.57 (95% CI 1.18–5.60), *p* = 0.014; T2 (3 m post-CRT): PFS HR = 2.88 (95% CI 1.21–6.83), *p* = 0.012	T2 (3 m post-CRT): OS HR = 3.67 (95% CI 1.41–9.55), *p* = 0.004	Baseline not prognostic; timing of clearance matters (earlier = better cCR). ctDNA complements PET-CT in distinguishing radiation esophagitis from residual disease.
Pinli Yue 2024 [37] China	38	Prospective RCT (socazolimab + chemo vs. placebo, then surgery); MRD-guided adjuvant; FUP 17 m	Tumor-informed panel (40 SNVs); sensitivity 0.001%; plasma	T0: 92% positive (not predictive of pCR); pre-surgery MRD (Tb)+: 73.7% (28/38); all 10 Tb- were pCR; postop MRD (Tp)+: 33.3% (11/33)	Pre-surgery MRD (Tb): Sens 100%, Spec 91%, *p* < 0.0001; postop MRD selects patients benefiting from adjuvant (MRD+ and adjuvant: 7/7 progression-free)	Within MRD+ (Tp) patients, adjuvant vs. no adjuvant: PFS HR = 0.032 (95% CI 0.003–0.390), *p* = 0.007; pathology alone: NS	NR	Pre-surgery MRD identifies residual disease (100% sensitivity). Postop MRD selects adjuvant-therapy beneficiaries; ctDNA adds prognostic information beyond pathology alone.
Xin Wang 2022 [38] China	40	Prospective; definitive CRT +/− esophagectomy; FUP 20.6 m; recurrence 48%	Panel NGS (cfDNA); top mutations TP53 85.7%, PRSS3 21.4%; 4 serial timepoints; plasma	T0: 70% positive (28/40; not prognostic); T1 (wk 4 CRT): 42.4% positive; T2 (1–3 m post-CRT): 29.6% positive; T3 (3–6 m post-CRT): 23.8% positive	T0: not predictive; rising T1 to T2 (high-risk pattern): PFS HR = 9.09 (95% CI 1.40–59.27); OS HR = 10.25 (95% CI 1.56–67.15)	T1 (wk 4; MV independent): PFS HR = 3.35 (95% CI 1.10–10.22), *p* = 0.03; T2: PFS HR = 5.45 (95% CI 1.72–17.26); T3: PFS HR = 5.83 (95% CI 1.53–22.22)	T2: OS HR = 4.02 (95% CI 1.27–12.75); T3: OS HR = 5.74 (95% CI 1.24–26.69)	Baseline is not prognostic. T1 (wk 4) is the first independent prognostic timepoint; HR escalates T1 to T3. A rising T1-to-T2 trajectory carries a very poor prognosis.
(**b**)								
**Study/Year**	**N**	**Setting & Design**	**ctDNA Method**	**Key Timepoint & Positivity Rate**	**Sensitivity/Specificity, PPV/NPV**	**DFS/RFS HR (95% CI), *p***	**OS HR (95% CI), *p***	**Key Finding/Lead Time**
Schoofs K. 2024 [40] Belgium	33	Prospective observational; feasibility/proof-of-concept; nCRT, then surgery (CROSS, *n* = 30); recurrence 57.6% (19/33)	cfRRBS (methylation-based); tumor-agnostic (no tumor tissue); CNV profiles; plasma; 7 timepoints	Median tumor fraction at t0: 2.2% (range 0–22.6%); cut-off: >2.6% (max healthy donor); tumor-fraction spike at t2 (4–6 d post-surgery): *p* = 0.002; tumor fraction >15% in 4/6 near clinical metastasis	~Sens 66.7% (informal: 4/6 near metastasis); Spec not calculated; PPV/NPV NR feasibility only—no formal accuracy analysis	Not formally analyzed (feasibility study)	Not formally analyzed	Methylation-based, tumor-agnostic cfDNA in EAC—no tumor tissue needed. Post-surgery tumor-fraction spike at 4–6 d is a trauma artifact. CNV: MYC, KRAS, EGFR, NOTCH2 amplifications detected.
Ococks E. 2021 [41] UK (OCCAMS) (Ann Oncol)	97	Prospective national cohort (OCCAMS); neoadjuvant chemo, then surgery (99%); serial cfDNA (245 samples); FUP 32.9 m; all cT3/T4; recurrence 47% (36/77)	AVENIO (Roche) 77-gene pan-cancer; tumor-agnostic; depth 7082×; CHIP correction (WBC sequenced; CHIP in 23%)	Pre-surgery: 49% positive (37/75); median VAF 0.52%; postop without CHIP: 21% positive (16/77); postop with CHIP: 16% positive (10/63); recurrence 9/10 (90%)	With CHIP correction: Sens 35%, Spec 97%, PPV 90%, NPV 68%, LR 12; CHIP present in 23% (mandatory WBC correction)	Without CHIP: DFS HR = 2.35 (95% CI 1.18–4.72), *p* = 0.013; with CHIP: DFS HR = 5.35 (95% CI 2.10–13.63), *p* = 0.001; MV (independent): HR = 4.77 (95% CI 1.93–11.8), *p* < 0.001	Without CHIP: CSS HR = 2.32 (95% CI 1.14–4.73), *p* = 0.017; with CHIP: CSS HR = 5.55 (95% CI 2.42–12.71), *p* = 0.0003	CHIP correction doubles the HR (CSS 2.32 to 5.55); TP53 is the most common CHIP variant and EAC driver, which makes WBC sequencing mandatory. SMAD4 enriched at recurrence. Low sensitivity (35%) reflects the non-EAC-specific panel.
Ococks E. 2021 [42] UK (OCCAMS) (Gastroenterology)	20	Retrospective OCCAMS subset (same cohort); tumor-informed vs. agnostic comparison; FUP up to 75+ months; 5/17 recurred	Signatera (Natera); tumor-informed; WGS 73× (tumor)/37× (blood) → 16 patient-specific SNVs → mPCR-NGS; median postop VAF+ 0.01% (0.001–15.9%)	Pre-surgery: 64.7% positive (11/17); all who recurred were ctDNA+ at baseline (Sens 100%, *p* < 0.0001); postop: 23.5% positive (4/17)	Postop: Sens 80% (4/5; 100% with strict criteria), Spec 100% (12/12), PPV 100%, NPV 92.3%; sensitivity doubles vs. tumor-agnostic (80% vs. 35%)	Preop+: median DFS 32.0 vs. 63.0 m (*p* = 0.042); postop+: median DFS 14.2 vs. 51.2 m (*p* < 0.0001); (HR not reported; small N); ctDNA- preop: 0/6 relapsed	Postop+: median CSS 18.0 vs. 53.4 m (*p* = 0.003); (HR not reported; small N)	First tumor-informed study in resected EAC; sensitivity doubles vs. agnostic (80% vs. 35%), Spec 100%. Late peritoneal recurrence (>4 yr) in 1 ctDNA- patient. Lead time ~1 yr (median 278 days; max >500 days).
Hofste L.S.M. 2022 [43] Netherlands	78	Prospective observational; locally advanced esophageal (cT2–3N+/cT4N0); all CROSS, then esophagectomy; no adjuvant; FUP PFS 28 m/OS 30 m; EAC 87%, ESCC 9%	Tumor-informed ultradeep hybrid-capture NGS; 15-gene panel (117 kb) + 56 MSI markers; depth 48,680× raw; threshold ≥ 4 mutant molecules + VAF > LoD; 22 healthy-donor normals	T0 (pre-CRT): 56.4% positive (44/78)—tumor burden marker; T1 (~day 11 CRT): not prognostic; T2 (preop, post-CRT): 10.3% positive (8/78)—key timepoint; 10/88 (11.4%) excluded (no panel mutations)	Sens/Spec NR (prognostic study); T2 ctDNA+ detected in pCR patients who later developed distant metastases (occult-disease marker)	T0: NS; T1: NS (too early); T2 (independent MV): PFS HR = 2.8 (95% CI 1.1–6.8), *p* = 0.03 (controlling for ypN+); UV: HR = 2.6 (95% CI 1.1–6.3), *p* = 0.04	T0: NS; T1: NS; T2 (independent MV): OS HR = 2.9 (95% CI 1.2–7.1), *p* = 0.02; UV: HR = 3.1 (95% CI 1.3–7.6), *p* = 0.01	Largest post-CRT ctDNA cohort in locally advanced esophageal cancer. T0 = tumor burden marker; T1 (~day 11) too early; T2 = key independent predictor, detected in pCR patients who later developed distant metastases. Lead time is not reported.
(**c**)								
**Study/Year**	**N**	**Setting & Design**	**ctDNA Method**	**Key Timepoint & Positivity Rate**	**Sensitivity/Specificity, PPV/NPV**	**DFS/RFS HR (95% CI), *p***	**OS HR (95% CI), *p***	**Key Finding/Lead Time**
Bai L. 2025 [44] China	37	Prospective; curative surgery (all stage III); FUP 14.6 m; sample = peritoneal lavage fluid (not blood plasma)	769-gene NGS; tumor-informed; sample = PLF (not plasma); CTCs also analyzed	PLF preop+: 68.6% (24/35); PLF postop+: 65.7% (23/35); CTCs preop+: 7/35; CTCs postop+: 17/35	Preop PLF: Sens 100%, Spec 42%; combined ctDNA + CTCs: strongest predictor for peritoneal metastasis	Peritoneal metastasis recurrence: preop PLF HR = 4.82 (95% CI 1.03–22.54); postop PLF HR = 4.83 (95% CI 1.03–22.54); combined ctDNA + CTCs: preop HR = 8.07 (95% CI 0.96–67.51); postop HR = 18.14 (95% CI 3.27–100.70), *p* = 0.0002	OS HR NR (peritoneal metastasis recurrence was the primary endpoint)	Unique use of peritoneal lavage fluid (not blood); predicts peritoneal metastatic recurrence. Combined ctDNA + CTCs markedly improves prediction (AUC 0.86 to 0.93). Limited specificity (42%).
Liu Z. 2025 [45] China	59	Prospective; curative gastrectomy +/− perioperative chemo; FUP 24.9 m	AVENIO 197-gene; tumor-informed; CHIP filtered; cut-off AF ≥0.2%; plasma	Preop+: 80.4% (45/56); postop+: 73.2% (30/41 evaluable); combined model (ctDNA + CEA + CA19-9 + CA72-4): AUC 0.940 for 3 yr survival (*p* = 0.002)	Postop MRD (1.115% cut-off): Sens 50%, Spec 90%; preop ctDNA: NS on MV; DCAF4L2 mutation: poor prognosis	Postop (1 mo)+: PFS HR = 3.85, *p* = 0.011 (KM); independent on MV (continuous ctDNA): HR = 4.00 (95% CI 1.30–12.0), *p* = 0.014	Postop (1 mo)+: OS HR = 5.30, *p* = 0.0022 (KM); combined model (ctDNA + CEA + CA19-9 + CA72-4): AUC = 0.940 for 3 yr survival	197-gene panel (largest GC review). Postop ctDNA is an independent predictor (MV). Combined ctDNA + tumor markers AUC 0.940—strongest model in GC literature. CHIP correction applied; DCAF4L2 a novel prognostic mutation.
Yuan S.Q. 2023 [46] China	100	Prospective; curative surgery; FUP 52.2 m (longest); stage II 37, III 63; recurrence 33/100	425-gene NGS (GeneseeqPrime); tumor-informed; threshold VAF ≥ 2%; plasma; 3 timepoints	Preop+: 33% (33/100); postop+: 25% (25/100); post-ACT+: 24.4% (10/41)	Post-ACT ctDNA (strongest timepoint): Sens 77.8%, Spec 90.6%; ERBB4 mutation: relapse even in ctDNA- patients	Preop+: RFS HR = 1.10 (95% CI 0.55–2.22), *p* = 0.785 (NS); postop+: RFS HR = 2.74 (95% CI 1.37–5.48), *p* = 0.003; post-ACT+: RFS HR = 14.99 (95% CI 3.08–72.96), *p* < 0.001 (strongest timepoint in GC review)	Preop+: OS HR = 1.13 (95% CI 0.53–2.43), *p* = 0.754 (NS); postop+: OS HR = 2.53 (95% CI not reported; KM); post-ACT+: OS HR = 11.88 (95% CI not reported; KM), *p* < 0.05	Longest FUP in GC (52.2 m). Preop ctDNA is not significant; post-ACT ctDNA is the strongest predictor. ERBB4 mutation predicts recurrence even in ctDNA-negative patients.
Zhou H. 2023 [47] China	14	Prospective; curative surgery; stages III–IV; *n* = 8 recurrences (very small N)	680-gene NGS (HapOncoCDx); tumor-informed; threshold VAF ≥ 5%; plasma; serial postop	Postop+: 57.1% (8/14); stage IV: 6/8 (75%) positive; stage III: 2/6 (33%) positive	Sens/Spec NR (too small N) 6/8 ctDNA+ patients progressed; CBLB mutation: ~14-fold worse prognosis (*p* < 0.01)	Postop+: PFS HR = 3.578 (95% CI 0.894–13.14), *p* = 0.037	Postop+: OS HR = 2.931 (95% CI 0.557–14.34), *p* = 0.203 (NS; small N)	Smallest GC series (*n* = 14). Postop ctDNA+ significantly predicts PFS despite small N; OS NS due to underpowering. CBLB mutation (15% of cohort vs. <2% in TCGA) a novel adverse prognostic marker.
Yang J. 2020 [48] China	46	Prospective; curative surgery; FUP 29.1 m; stages I–III; recurrence 19/46	1021-gene panel (1.09 Mb); tumor-informed; matched PBMC normal control; serial postop; plasma	Preop+: 45.5% (20/44); postop+: 18.4% (7/38)	Preop ctDNA+ associated with stage (68% of stage III cases ctDNA+); postop+: 7/7 (100%) recurred vs. 32% (*p* = 0.0015); postop: Sens 39%, Spec 100%	Postop+: DFS HR = 6.56 (95% CI not reliably reported), *p* < 0.0001; longitudinal any postop+: DFS HR = 14.78 (95% CI 7.99–61.29), *p* < 0.0001; median DFS+: 216 days vs. NR	Postop+: OS HR = 5.96 (95% CI 3.77–138.1), *p* = 0.0007; longitudinal+: OS HR = 7.66 (95% CI 2.92–21.06), *p* = 0.002	Postop ctDNA+: 100% recurrence (7/7); longitudinal DFS HR 14.78 among highest in GC. Specificity is 100%. Lead-time median 179 days (~6 months) before imaging.
Leal A. 2020 [49] NL/SE/DK (CRITICS)	50	Prospective (CRITICS RCT substudy); curative surgery; FUP 42 m; perioperative ECX vs. ECX + CRT	TEC-seq; 58 cancer-driver genes (81 kb); >30,000×; tumor-agnostic; WBC DNA filter essential (CHIP)	Baseline+: 54% (27/50); preop (after 3 cycles) +: 63.3% (19/30); postop MRD+: 45% (9/20); all 11 MRD- alive/disease-free	Without WBC filter: *p* = 0.76 (NS)—CHIP makes ctDNA non-informative; with WBC filter: preop EFS HR = 3.0; postop MRD HR = 21.8	Preop (after 3 cycles) +: EFS HR = 3.0 (95% CI 1.3–6.9), *p* = 0.012; postop MRD+: EFS HR = 21.8 (95% CI 3.9–123.1), *p* < 0.001	Preop (after 3 cycles) +: OS HR = 2.7 (95% CI 1.1–6.7), *p* = 0.030; postop MRD+: OS HR = 21.8 (95% CI 3.9–123.1), *p* < 0.001	Postop MRD HR = 21.8 (EFS and OS identical)—highest in GC cohort. Without WBC filtering, ctDNA is non-significant (*p* = 0.76), which makes WBC correction mandatory. All 11 MRD- patients disease-free at 42 m. Lead time 8.9 months.
Kim Y.W. 2019 [50] Korea	19	Retrospective; curative surgery; FUP 12 m; stages II–IV; 6/25 (24%) non-shedders excluded	WGS (30×) → personalized chromosomal rearrangements → PCR + ddPCR; plasma	Preop+: 57.9% (11/19)—not correlated with recurrence (*p* = 0.6372); postop+: 42.1% (8/19)—associated with recurrence (*p* = 0.0023)	Preop ctDNA+: not correlated (*p* = 0.6372); postop ctDNA+: *p* = 0.0023; Sens/Spec NR (small N)	Preop+: RFS NS (*p* = 0.6372); postop+: *p* = 0.0023 (HR not stated; small N)	OS: NR	Only WGS-based chromosomal-rearrangement approach in GC. Preop ctDNA not predictive; postop ctDNA strongly predicts recurrence. Lead time 4.05 months; 24% non-shedders excluded.
Cabel L. 2019 [51] France	32	Prospective proof-of-concept; curative surgery; FUP 26 m; perioperative FOLFOX +/− trastuzumab; pCR (ypT0N0) 7 (22%)	Tumor-informed ddPCR (per patient); 39-gene NGS on tumor → customized ddPCR; MAF threshold > 0.1%; plasma	Baseline+: 21% (4/19 evaluable), 12/32 (37.5%) had no trackable mutation (excluded); diffuse subtype: 0/6 positive; after preop chemo: 0/18 (0%) detectable; postop: 7.7% positive (1/13)	Baseline ctDNA: not predictive of relapse (*p* = 0.52); lowest sensitivity in entire GC cohort	Baseline+: RFS NS (*p* = 0.52); postop+: 1 patient detected, relapsed at 3 m (HR NR; very small ctDNA+ group)	OS: NR	Lowest sensitivity in the GC cohort. Key limitations: 37.5% had no trackable mutation (panel too small); diffuse subtype 0/6 positive; after preop chemo 0/18 detectable. Important negative study.
(**d**)								
**Study/Year**	**N**	**Setting & Design**	**ctDNA Method**	**Key Timepoint & Positivity Rate**	**Sensitivity/Specificity, PPV/NPV**	**DFS/RFS HR (95% CI), *p***	**OS HR (95% CI), *p***	**Key Finding/Lead Time**
Zaanan A. 2025 [52] France (PLAGAST)	62	Prospective; curative surgery; FUP 29 m; neoadjuvant 89% FLOT-based (chemo +/− ICI); recurrence 47%; mixed GEJ/GC	Signatera (Natera); tumor-informed; WES + 16-plex mPCR-NGS; cut-off ≥2 SNVs; plasma; 4 timepoints	Pre-NAT+: 69.6% (39/56); during NAT+: 51.2% (21/41); post-NAT MRD+: 26.8% (11/41); post-NAT MRD+: 100% recurrence (7/7); 24 m RFS 0% vs. 62.8% (MRD-)	Combined MRD+/ypN+: HR = 384.99 (RFS); C-index RFS 0.87, OS 0.91; TRG 4/5: all 7 persistently ctDNA+	During NAT+: RFS HR = 6.17 (95% CI 1.99–19.12), *p* = 0.002; post-NAT+: RFS HR = 5.26 (95% CI 1.96–14.12), *p* = 0.001; postop MRD+ (independent): RFS HR = 12.94 (95% CI 4.23–39.59), *p* < 0.0001	During NAT+: OS HR = 4.71 (95% CI 1.24–17.86), *p* = 0.022; post-NAT+: OS HR = 7.35 (95% CI 2.35–22.95), *p* = 0.001; postop MRD+: OS HR = 14.54 (95% CI 4.54–46.6), *p* < 0.0001	Postop MRD HR = 14.54—highest in mixed GI. Post-NAT MRD+: 100% recurrence (7/7). Combined MRD+/ypN+ HR = 384.99; C-index 0.87/0.91. ctDNA correlates with TRG. Lead time 184 days (longest in mixed cohort).
Hu Q. 2025 [53] Japan (Kyushu Univ)	40	Two-step observational (retrospective pilot *n* = 6 + prospective *n* = 34); curative esophagectomy +/− NAC; FUP 22.6/13.9 m; ESCC 90%, EAC 10%	In-house 250-gene NGS; tumor-informed (coding + non-coding); novel definition: ctDNA+ = increase vs. pre-therapy baseline (kinetics-based, not absolute threshold); matched buffy-coat germline filtering; 6 timepoints	Pre-therapy: 100% positive (not predictive); initial postop+: 50% (20/40); recurrence ctDNA+: 10/20 (50%) vs. ctDNA-: 1/20 (5%); 4 ctDNA- patients converted to ctDNA+ during FUP	Postop: Sens 90.9%, Spec 65.5%, AUC 0.77; 18 m PFS: ctDNA+ 53.3% vs. ctDNA- 95.0%	All patients PFS: HR = 12.6 (95% CI 1.6–99.0), *p* = 0.002; R0 patients RFS: HR = 11.1 (95% CI 1.4–89.0), *p* = 0.006; MV (independent): HR = 19.1 (95% CI 2.21–164.85), *p* = 0.007 (wide CI; small N)	OS HR NR (PFS/RFS primary endpoints)	Novel kinetics-based definition (increase vs. baseline). MV HR = 19.1 (independent). 4 initially ctDNA-negative patients converted to ctDNA+ during surveillance, which underscores the value of serial monitoring. The lead time is 90 days.
Iden C.R. 2025 [54] Denmark	86	Prospective; curative surgery; FUP 26.7 m; perioperative chemo; 41 recurrences; cT3/T4 62.8%; mixed EAC/GEJ/GC	ddPCR TriMeth (C9orf50, KCNQ5, CLIP4 methylation); tumor-agnostic (no tumor tissue); cut-off ≥2 of 3 markers; plasma; 4 timepoints	Preop+: 55.7% (44/79); after 1 cycle+: 37% (27/73); MRD window+ (postop): 8/53 (15%); 24 mo RFS ctDNA+ 12.5% vs. ctDNA- 70.7%	Recurrence Sens/Spec not formally reported (detection-rate–based; see Positivity column)	After 1 cycle+: RFS HR = 2.54 (95% CI 1.33–4.85), *p* = 0.005; after surgery (MRD)+: RFS HR = 6.22 (95% CI 2.39–16.2), *p* < 0.001	After 1 cycle+: OS HR = 2.23 (95% CI 1.07–4.62), *p* = 0.032; after surgery+: OS HR = 6.37 (95% CI 2.10–19.3), *p* = 0.001; MV independent: HR = 7.33 (95% CI 2.39–22.47), *p* < 0.001	Only tumor-agnostic methylation platform in mixed upper GI. Postoperative MRD the strongest prognostic timepoint (independent OS HR 7.33). Early on-treatment (cycle 1) ctDNA also prognostic, offering response information before imaging.
Lander E.M. 2024 [55] USA (11 sites)	42	Retrospective real-world multi-center; curative surgery; FUP 28.5 m; pCR/near-pCR only (TRG-0/1); mixed EAC/GEJ/GC	Signatera (Natera); tumor-informed; WES + 16-plex mPCR-NGS; cut-off ≥2 SNVs; plasma; 2 windows (MRD ≤ 16 wk; surveillance > 16 wk)	MRD window: 13% positive (3/23); recurrence 2/3 (67%) vs. 3/20 (15%); surveillance: 15.6% positive (5/32); recurrence 5/5 (100%) vs. 2/27 (7.4%)	MRD window+: recurrence 67% vs. 15%; surveillance+: 100% recurrence (5/5)	MRD window+: RFS HR = 6.2 (95% CI 1.0–37.6), *p* = 0.049; surveillance+: RFS HR = 37.6 (95% CI 4.3–325.6), *p* < 0.001 (highest RFS HR in review)	OS HR NR	Restricted to pCR/near-pCR patients: ctDNA detects residual disease even after pathological complete response. Surveillance HR = 37.6 (highest RFS HR in review); surveillance ctDNA+ = 100% recurrence. The lead time is 78 days.
Huffman B.M. 2022 [56] USA (>70 sites)	295	Retrospective real-world multi-center; curative surgery; FUP 13.9 m; largest real-world upper GI ctDNA study; stages I–IV; mixed EAC/GEJ/GC	Signatera (Natera); tumor-informed; WES + 16-plex mPCR-NGS; cut-off ≥2 SNVs; plasma; 4 timepoints	Preop+: 95.8% (23/24); MRD window+: 23.5% (16/68)	Postop anytime: Sens 85.7%, Spec 95.5%; surveillance: Sens 80%, Spec 98.3%; very low false-positive rate	MRD window+ (independent MV): RFS HR = 10.7 (95% CI 4.3–29.3), *p* < 0.0001; anytime postop+: HR = 23.6 (95% CI 10.2–66.0); surveillance+: HR = 17.7 (95% CI 7.3–50.7); MV HR = 11.82 (95% CI 6.18–22.6), *p* < 0.001	OS HR NR (RFS primary endpoint)	Largest series in review (*n* = 295, >70 sites). HR escalation: MRD window 10.7, anytime postop 23.6, surveillance 17.7. Specificity 95.5–98.3% (very low false-positive rate). Lead time is not reported.

Abbreviations: ACT = adjuvant chemotherapy; AUC = area under the curve; cfDNA = cell-free DNA; CRT = chemoradiotherapy; CSS = cancer-specific survival; DFS = disease-free survival; ddPCR = droplet digital PCR; EAC = esophageal adenocarcinoma; EFS = event-free survival; ESCC = esophageal squamous cell carcinoma; FUP = follow-up; GC = gastric cancer; GEJ = gastroesophageal junction; HR = hazard ratio; MAF = mutant allele frequency; MIE = minimally invasive esophagectomy; MRD = minimal residual disease; mPCR = multiplex polymerase chain reaction; MV = multivariable; NAC = neoadjuvant chemotherapy; NAT = neoadjuvant therapy; nCRT = neoadjuvant chemoradiotherapy; NGS = next-generation sequencing; NPV = negative predictive value; NR = not reported; NS = not significant; pCR = pathological complete response; PLF = peritoneal lavage fluid; PPV = positive predictive value; RFS = recurrence-free survival; Sens = sensitivity; SNV = single nucleotide variant; Spec = specificity; TRG = tumor regression grade; UV = univariate; VAF = variant allele frequency; WBC = white blood cell; WES = whole-exome sequencing; WGS = whole-genome sequencing.

**Table 4:** Postoperative ctDNA sampling parameters across included studies with postoperative minimal residual disease (MRD) assessment.

Study (Year)	Surgery → Postoperative Blood Collection	Timing Relative to Adjuvant Therapy	Serial Postoperative Timepoints	Evaluable Postoperative Samples	ctDNA Lead Time Before Recurrence
Esophageal squamous cell carcinoma (ESCC)					
Fang C.Y. 2025 [30]	7–14 days	None (surgery-only cohort)	Single	60/125 positive	Not reported
Jimin Li 2025 [31]	1 month (±1 wk)	Adjuvant in 29/35 (timing not specified)	Single	6/35 positive	Not reported
Rentong Gu 2025 [32]	Day 7	Before adjuvant (adjuvant in 20/54)	Single	TP53 6/54; PIK3CA 6/54	Not reported
Heng Jiao 2025 [33]	≤2 months (landmark, post-R0)	Neoadjuvant → surgery → adjuvant	Landmark + serial follow-up	11/43 positive	Not reported
Ko J.M.Y. 2025 [34]	0–1 month (first of 7 windows)	Surveillance windows; adjuvant in 19	7 postoperative windows (0–1 mo → 1.5–2 yr)	6/47 positive	158 d (from pre-surgery sample) ‡
Pinli Yue 2024 [37]	1 month (Tp)	Before adjuvant (used to guide it)	Single	11/33 positive	Not reported
Liu T. 2021 [39]	1 week (surgical-trauma window)	Non-adjuvant subgroup primary; adjuvant in 19	Pre- + postop	6/38 positive	Not reported
Esophageal adenocarcinoma (EAC)					
Schoofs K. 2024 [40]	4–6 days, then up to >12 mo	Given to some (feasibility)	5 postoperative (t2–t6)	33 (feasibility)	Not analyzed (feasibility)
Ococks E. 2021 [41] (Ann Oncol)	Serial post-surgery (to >2000 d)	Offered routinely post-surgery	Serial (116 postop samples)	16/77; 10/63 (CHIP-corrected)	Recurrence in 9/10 positive
Ococks E. 2021 [42] (Gastroenterology)	≥1 postop sample/patient, serial	Perioperative	Serial	4/17 positive	278 days (max > 500)
Gastric cancer (GC)					
Bai L. 2025 [44]	Intraoperative peritoneal lavage fluid (post-resection)	Intraoperative (precedes adjuvant)	Single (PLF)	23/35 PLF positive	Not reported
Liu Z. 2025 [45]	1 month	Not specified (adjuvant in ~61%)	1 mo, then q3mo	30/41 positive	Not reported
Yuan S.Q. 2023 [46]	≤1 wk (median 4 d); + post-ACT ≤ 3 mo	Both (before and after adjuvant chemo)	2 (postop; post-ACT)	25/100 postop; 10/41 post-ACT	Not reported
Zhou H. 2023 [47]	4–6 weeks	Before adjuvant chemotherapy	4–6 wk + serial in 5 patients	8/14 positive	Not reported
Yang J. 2020 [48]	1 month (9–48 d), then q3mo/q6mo	Both (before and after adjuvant chemo)	1 mo → q3mo (yr 1) → q6mo	7/38 positive	179 days
Leal A. 2020 [49]	Median 6.5 weeks	Before adjuvant (not sampled after)	Single	9/20 positive	8.9 months
Kim Y.W. 2019 [50]	1 month	Not specified	5 (1, 3, 6, 9, 12 mo)	8/19 positive	4.05 months
Cabel L. 2019 [51]	<1 month	Before resumed postoperative chemo	Single (<1 mo)	1/13 positive	Not reported
Mixed upper gastrointestinal					
Zaanan A. 2025 [52] (PLAGAST)	MRD window 2–12 wk (median 41 d)	Before adjuvant	Single (MRD window)	10/50 positive (47/50 analyzed)	184 days
Hu Q. 2025 [53]	1 month (median 1.2 mo)	Not specified	4 postoperative (1, 3, 6, 12 mo)	20/40 positive	90 days
Iden C.R. 2025 [54]	4–6 weeks	Not stated relative to adjuvant	Single	8/53 positive	Not reported
Lander E.M. 2024 [55]	MRD window ≤ 16 wk; surveillance > 16 wk	Window before adjuvant; surveillance after	No fixed schedule (clinician discretion)	3/23 (window); 5/32 (surveillance)	78 days
Huffman B.M. 2022 [56]	MRD window ≤ 16 wk (before systemic Rx); surveillance ≥ 2 wk after end of Rx	Window before adjuvant; surveillance after	No fixed schedule (clinician discretion)	16/68 (MRD window)	Not reported (not estimable)

Postoperative sampling parameters as reported in each primary study. ‡ For Ko et al., the 158-day lead time derives from a pre-surgery (end of neoadjuvant chemoradiotherapy) sample rather than a postoperative sample. Bai et al. used intraoperative peritoneal lavage fluid (PLF) rather than plasma. ACT = adjuvant chemotherapy; CHIP = clonal hematopoiesis; MRD = minimal residual disease; PLF = peritoneal lavage fluid; q3mo/q6mo = every 3/6 months; Rx = systemic therapy; Tp = postoperative timepoint.

## Data Availability

The original contributions presented in this study are included in the article and Supplementary Materials. Further inquiries can be directed to the corresponding author.

## Supplementary Materials

The following supporting information can be downloaded at: https://www.mdpi.com/article/10.3390/jcm15166222/s1. File S1: Search Strategy Overview; Table S1: Newcastle–Ottawa Scale (NOS) quality assessment of included studies; Table S2: completed PRISMA-ScR checklist.
