# Supplementary material for: Circulating Tumor DNA for Minimal Residual Disease Detection and Recurrence Prediction in Upper Gastrointestinal Cancers: A Scoping Review
Source: J Clin Med. 2026 Aug 11;15(16):6222. doi: 10.3390/jcm15166222 (PMC13513700; doi:10.3390/jcm15166222)
Supplement: Supplementary file 1 [file jcm-15-06222-s001.zip › Supplementary Table S2.pdf]

Supplementary Table S2. Completed PRISMA-ScR Checklist

Preferred Reporting Items for Systematic reviews and Meta-Analyses extension for Scoping Reviews (PRISMA-ScR)

Manuscript: "Circulating Tumor DNA for Minimal Residual Disease Detection and Recurrence Prediction in Upper Gastrointestinal Cancers: A Scoping Review."

The "Reported on" column indicates the section/sub-section of the manuscript where each item is addressed. Item numbering follows the original PRISMA-ScR checklist (Tricco et al., 2018 [26]).

Section	Item	PRISMA-ScR Checklist Item	Reported on (location in manuscript)
**TITLE**			
Title	1	Identify the report as a scoping review.	Title page (the title states "A Scoping Review").
**ABSTRACT**			
Structured summary	2	Provide a structured summary that includes (as applicable) background, objectives, eligibility criteria, sources of evidence, charting methods, results, and conclusions that relate to the review questions and objectives.	Abstract (Background, Methods, Results, Conclusions).
**INTRODUCTION**			
Rationale	3	Describe the rationale for the review in the context of what is already known. Explain why the review questions/objectives lend themselves to a scoping review approach.	Introduction (rationale developed across the section).
Objectives	4	Provide an explicit statement of the questions and objectives being addressed with reference to their key elements (e.g., population or participants, concepts, and context) or other relevant key elements used to conceptualize the review questions and/or objectives.	Introduction (final paragraph); Methods, Section 2.1 (Study Design).
**METHODS**			
Protocol and registration	5	Indicate whether a review protocol exists; state if and where it can be accessed (e.g., a Web address); and if available, provide registration information, including the registration number.	Methods, Section 2.1 — the review was not registered in a public registry and no protocol was published a priori.
Eligibility criteria	6	Specify characteristics of the sources of evidence used as eligibility criteria (e.g., years considered, language, and publication status), and provide a rationale.	Methods, Section 2.2 (Eligibility Criteria).
Information sources	7	Describe all information sources in the search (e.g., databases with dates of coverage and contact with authors to identify additional sources), as well as the date the most recent search was executed.	Methods, Section 2.3 (PubMed, Scopus, and the Cochrane Library; last searched 3 April 2026).
Search	8	Present the full electronic search strategy for at least 1 database, including any limits used, such that it could be repeated.	Methods, Section 2.3; Table 1 (search strategy overview); full search algorithms in Supplementary File S1.
Selection of sources of evidence	9	State the process for selecting sources of evidence (i.e., screening and eligibility) included in the scoping review.	Methods, Section 2.4 (Study Selection).
Data charting process	10	Describe the methods of charting data from the included sources of evidence (e.g., calibrated forms or forms that have been tested by the team before their use, and whether data charting was done independently or in duplicate) and any	Methods, Section 2.5 (Data Extraction — two independent reviewers; pre-piloted Excel charting form).

Section	Item	PRISMA-ScR Checklist Item	Reported on (location in manuscript)
		processes for obtaining and confirming data from investigators.	
Data items	11	List and define all variables for which data were sought and any assumptions and simplifications made.	Methods, Section 2.5 (list of charted variables).
Critical appraisal of individual sources of evidence	12	If done, provide a rationale for conducting a critical appraisal of included sources of evidence; describe the methods used and how this information was used in any data synthesis (if appropriate).	Methods, Section 2.6 (Newcastle–Ottawa Scale); Supplementary Table S1. [Optional item — addressed.]
Summary measures	13	*Not applicable for scoping reviews.*	—
Synthesis of results	14	Describe the methods of handling and summarizing the data that were charted.	Methods, Section 2.7 (Data Synthesis — narrative synthesis grouped by tumor type and clinical context).
Risk of bias across studies	15	*Not applicable for scoping reviews.*	—
Additional analyses	16	*Not applicable for scoping reviews.*	—
**RESULTS**			
Selection of sources of evidence	17	Give numbers of sources of evidence screened, assessed for eligibility, and included in the review, with reasons for exclusions at each stage, ideally using a flow diagram.	Results, Section 3.1; Figure 1 (PRISMA-ScR flow diagram).
Characteristics of sources of evidence	18	For each source of evidence, present characteristics for which data were charted and provide the citations.	Results, Section 3.2; Tables 2a–2d.
Critical appraisal within sources of evidence	19	If done, present data on critical appraisal of included sources of evidence (see item 12).	Methods, Section 2.6; Results, Section 3.2; Supplementary Table S1. [Optional item — addressed.]
Results of individual sources of evidence	20	For each included source of evidence, present the relevant data that were charted that relate to the review questions and objectives.	Results, Section 3.3 (3.3.1–3.3.4); Table 3(a)–(d).
Synthesis of results	21	Summarize and/or present the charting results as they relate to the review questions and objectives.	Results, Section 3.3; Discussion, Section 4.1.
Risk of bias across studies	22	*Not applicable for scoping reviews.*	—
Additional analyses	23	*Not applicable for scoping reviews.*	—
**DISCUSSION**			
Summary of evidence	24	Summarize the main results (including an overview of concepts, themes, and types of evidence available), link to the review questions and objectives, and consider the relevance to key groups.	Discussion, Sections 4.1–4.3.
Limitations	25	Discuss the limitations of the scoping review process.	Discussion, Section 4.4 (Limitations).
Conclusions	26	Provide a general interpretation of the results with respect to the review questions and objectives, as well as potential implications and/or next steps.	Discussion, Section 4.5 (Future Directions); Conclusions (Section 5).
**FUNDING**			
Funding	27	Describe sources of funding for the included sources of evidence, as well as sources of funding for the scoping review. Describe the role of the funders of the scoping review.	Funding statement (this research received no external funding).

Notes.

Items 12 and 19 (critical appraisal of, and within, individual sources of evidence) are optional in PRISMA-ScR; both were nonetheless addressed in this review using the Newcastle–Ottawa Scale (Methods, Section 2.6; Supplementary Table S1).

Items 13, 15, 16, 22, and 23 are marked by the PRISMA-ScR authors as “Not applicable for scoping reviews” and are retained here with their original PRISMA item numbers.

Reference: Tricco AC, Lillie E, Zarin W, et al. PRISMA Extension for Scoping Reviews (PRISMA-ScR): Checklist and Explanation. Ann Intern Med. 2018;169(7):467–473. doi:10.7326/M18-0850. (This reference is cited in the main text and listed in the reference list as reference [26].)
