# Supplementary material for: Circulating Tumor DNA for Minimal Residual Disease Detection and Recurrence Prediction in Upper Gastrointestinal Cancers: A Scoping Review
Source: J Clin Med. 2026 Aug 11;15(16):6222. doi: 10.3390/jcm15166222 (PMC13513700; doi:10.3390/jcm15166222)
Supplement: Supplementary file 1 [file jcm-15-06222-s001.zip › Supplementary File S1.pdf]

Circulating Tumor DNA for Minimal Residual Disease Detection and Recurrence Prediction in Upper Gastrointestinal Cancers: A Scoping Review

Supplementary File S1: Search Strategy Overview

Database	Field tags	Wildcard	Date searched
PubMed	[Mesh], [tw], [tiab]	* (truncation)	3 April 2026
Scopus	TITLE-ABS-KEY()	* (truncation)	3 April 2026
Cochrane	:ti,ab,kw + MeSH descriptors	* (truncation)	3 April 2026

Note: *All searches were limited to human studies and English-language publications. No date restrictions were applied at the search stage; date filtering was performed at the screening stage. The three blocks were combined using the Boolean operator AND.*

1. PubMed Search Algorithm

Block #1 — ctDNA / Liquid Biopsy

#1 — ctDNA / Liquid biopsy block

("Circulating Tumor DNA"[Mesh] OR "circulating tumor DNA"[tw]

OR "circulating tumour DNA"[tw] OR "ctDNA"[tw]

OR "cell-free DNA"[tw] OR "cell free DNA"[tw] OR "cfDNA"[tw]

OR "liquid biopsy"[tw] OR "minimal residual disease"[tw]

OR "molecular residual disease"[tw] OR "MRD"[tiab])

Block #2 — Recurrence / Survival

#2 — Recurrence / Survival block

("Neoplasm Recurrence, Local"[Mesh] OR "recurrence"[tw]

OR "tumor recurrence"[tw] OR "tumour recurrence"[tw]

OR "cancer recurrence"[tw] OR "relapse"[tw]

OR "disease recurrence"[tw] OR "postoperative recurrence"[tw]

OR "early recurrence"[tw] OR "metastatic recurrence"[tw]

OR "disease-free survival"[tw] OR "recurrence-free survival"[tw]

OR "relapse-free survival"[tw] OR "DFS"[tiab] OR "RFS"[tiab]

OR "progression-free survival"[tw] OR "PFS"[tiab])

Block #3 — Digestive System Neoplasms

#3 — Digestive System Neoplasms

("Digestive System Neoplasms"[Mesh]

OR "digestive system cancer*"[tw] OR "digestive system tumor*"[tw]

OR "digestive system tumour*"[tw] OR "digestive system malignanc*"[tw]

OR "gastrointestinal cancer*"[tw] OR "gastrointestinal tumor*"[tw]

OR "gastrointestinal tumour*"[tw] OR "gastrointestinal malignanc*"[tw]

OR "GI cancer*"[tw] OR "GI malignanc*"[tw]

OR "esophageal cancer"[tw] OR "oesophageal cancer"[tw]

OR "esophageal neoplasm*"[tw] OR "gastric cancer"[tw]

OR "stomach cancer"[tw] OR "gastric neoplasm*"[tw]

OR "small intestine cancer"[tw] OR "duodenal cancer"[tw]

OR "jejunum cancer"[tw] OR "ileal cancer"[tw]

OR "colorectal cancer"[tw] OR "colon cancer"[tw] OR "rectal cancer"[tw]

OR "colorectal neoplasm*"[tw] OR "anal cancer"[tw]

OR "pancreatic cancer"[tw] OR "pancreatic neoplasm*"[tw]

OR "pancreatic adenocarcinoma"[tw] OR "liver cancer"[tw]

OR "hepatic cancer"[tw] OR "hepatocellular carcinoma"[tw]

OR "HCC"[tiab] OR "cholangiocarcinoma"[tw]

OR "bile duct cancer"[tw] OR "biliary tract cancer"[tw]

OR "gallbladder cancer"[tw])

Final PubMed Query: #1 AND #2 AND #3

Note: [Mesh] = MeSH controlled vocabulary; [tw] = text word (title + abstract + other text fields); [tiab] = title and abstract; * = truncation operator.

2. Scopus Search Algorithm

Block #1 — ctDNA / Liquid Biopsy

#1 — ctDNA / Liquid biopsy block

(TITLE-ABS-KEY("circulating tumor DNA") OR TITLE-ABS-KEY("circulating tumour DNA")

OR TITLE-ABS-KEY("ctDNA") OR TITLE-ABS-KEY("cell-free DNA")

OR TITLE-ABS-KEY("cfDNA") OR TITLE-ABS-KEY("liquid biopsy")

OR TITLE-ABS-KEY("minimal residual disease")

OR TITLE-ABS-KEY("molecular residual disease") OR TITLE-ABS-KEY("MRD"))

Block #2 — Recurrence / Survival

#2 — Recurrence / Survival block

(TITLE-ABS-KEY("recurrence") OR TITLE-ABS-KEY("tumor recurrence")

OR TITLE-ABS-KEY("tumour recurrence") OR TITLE-ABS-KEY("cancer recurrence")

OR TITLE-ABS-KEY("relapse") OR TITLE-ABS-KEY("disease recurrence")

OR TITLE-ABS-KEY("postoperative recurrence")

OR TITLE-ABS-KEY("disease-free survival")

OR TITLE-ABS-KEY("recurrence-free survival")

OR TITLE-ABS-KEY("relapse-free survival") OR TITLE-ABS-KEY("DFS")

OR TITLE-ABS-KEY("RFS") OR TITLE-ABS-KEY("progression-free survival")

OR TITLE-ABS-KEY("PFS"))

Block #3 — Digestive System Neoplasms

#3 — Digestive System Neoplasms block

(TITLE-ABS-KEY("gastrointestinal cancer*")

OR TITLE-ABS-KEY("gastrointestinal tumor*")

OR TITLE-ABS-KEY("gastrointestinal tumour*")

OR TITLE-ABS-KEY("gastrointestinal malignancy")

OR TITLE-ABS-KEY("GI cancer*") OR TITLE-ABS-KEY("upper gastrointestinal")

OR TITLE-ABS-KEY("esophageal cancer") OR TITLE-ABS-KEY("oesophageal cancer")

OR TITLE-ABS-KEY("esophageal neoplasm*") OR TITLE-ABS-KEY("esophageal adenocarcinoma")

OR TITLE-ABS-KEY("esophageal squamous cell carcinoma")

OR TITLE-ABS-KEY("gastric cancer") OR TITLE-ABS-KEY("stomach cancer")

OR TITLE-ABS-KEY("gastric neoplasm*") OR TITLE-ABS-KEY("gastroesophageal junction")

OR TITLE-ABS-KEY("GEJ") OR TITLE-ABS-KEY("colorectal cancer")

OR TITLE-ABS-KEY("colon cancer") OR TITLE-ABS-KEY("rectal cancer")

OR TITLE-ABS-KEY("pancreatic cancer") OR TITLE-ABS-KEY("pancreatic adenocarcinoma")

OR TITLE-ABS-KEY("hepatocellular carcinoma") OR TITLE-ABS-KEY("HCC")

OR TITLE-ABS-KEY("cholangiocarcinoma") OR TITLE-ABS-KEY("biliary tract cancer")

OR TITLE-ABS-KEY("bile duct cancer") OR TITLE-ABS-KEY("gallbladder cancer"))

Final Scopus Query: #1 AND #2 AND #3

Note: *TITLE-ABS-KEY () searches title, abstract, and keywords simultaneously. * = truncation operator. Searches were conducted in Scopus Advanced Search.*

3. Cochrane Library Search Algorithm

Block #1 — ctDNA / Liquid Biopsy

#1 — ctDNA / Liquid biopsy block

(MeSH descriptor: [Circulating Tumor DNA] explode all trees

OR "circulating tumor DNA":ti,ab,kw OR "circulating tumour DNA":ti,ab,kw

OR ctDNA:ti,ab,kw OR "cell-free DNA":ti,ab,kw

OR cfDNA:ti,ab,kw OR "liquid biopsy":ti,ab,kw

OR "minimal residual disease":ti,ab,kw

OR "molecular residual disease":ti,ab,kw OR MRD:ti,ab,kw)

Block #2 — Recurrence / Survival

#2 — Recurrence / Survival block

(MeSH descriptor: [Neoplasm Recurrence, Local] explode all trees

OR recurrence:ti,ab,kw OR "tumor recurrence":ti,ab,kw

OR "tumour recurrence":ti,ab,kw OR relapse:ti,ab,kw

OR "disease recurrence":ti,ab,kw OR "postoperative recurrence":ti,ab,kw

OR "disease-free survival":ti,ab,kw

OR "recurrence-free survival":ti,ab,kw

OR "relapse-free survival":ti,ab,kw OR DFS:ti,ab,kw

OR RFS:ti,ab,kw OR "progression-free survival":ti,ab,kw OR PFS:ti,ab,kw)

Block #3 — Digestive System Neoplasms

#3 — Digestive System Neoplasms block

(MeSH descriptor: [Digestive System Neoplasms] explode all trees

OR "gastrointestinal cancer*":ti,ab,kw

OR "gastrointestinal tumor*":ti,ab,kw

OR "gastrointestinal tumour*":ti,ab,kw

OR "esophageal cancer":ti,ab,kw OR "oesophageal cancer":ti,ab,kw

OR "esophageal neoplasm*":ti,ab,kw

OR "esophageal squamous cell carcinoma":ti,ab,kw

OR "esophageal adenocarcinoma":ti,ab,kw

OR "gastric cancer":ti,ab,kw OR "stomach cancer":ti,ab,kw

OR "gastric neoplasm*":ti,ab,kw OR "gastroesophageal junction":ti,ab,kw

OR "colorectal cancer":ti,ab,kw OR "colon cancer":ti,ab,kw

OR "rectal cancer":ti,ab,kw OR "pancreatic cancer":ti,ab,kw

OR "hepatocellular carcinoma":ti,ab,kw OR HCC:ti,ab,kw

OR "cholangiocarcinoma":ti,ab,kw OR "biliary tract cancer":ti,ab,kw

OR "gallbladder cancer":ti,ab,kw)

Final Cochrane Query: #1 AND #2 AND #3

Note: *MeSH descriptor [explode all trees] includes all narrower terms. :ti,ab,kw searches title, abstract, and keywords. * = truncation operator. Searches were conducted in Cochrane Library Advanced Search.*
