# Supplementary material for: Circulating Tumor DNA for Minimal Residual Disease Detection and Recurrence Prediction in Upper Gastrointestinal Cancers: A Scoping Review
Source: J Clin Med. 2026 Aug 11;15(16):6222. doi: 10.3390/jcm15166222 (PMC13513700; doi:10.3390/jcm15166222)
Supplement: Supplementary file 1 [file jcm-15-06222-s001.zip › Supplementary Table S1.pdf]

Supplementary Table S1. Newcastle–Ottawa Scale (NOS) Quality Assessment of Included Studies

Study	Selection	Comparability	Outcome	Total NOS score	Quality
Fang C.Y. et al.	3	1	3	7/9	High
Jimin Li et al.	3	2	2	7/9	High
Rentong Gu et al.	3	2	3	8/9	High
Heng Jiao et al.	4	2	3	9/9	High
Ko J.M.Y. et al.	3	1	3	7/9	High
Yang Yang et al.	2	0	2	4/9	Moderate
Baoqing Chen et al.	2	0	3	5/9	Moderate
Pinli Yue et al.	2	0	2	4/9	Moderate
Xin Wang et al.	3	2	3	8/9	High
Liu T. et al.	2	0	2	4/9	Moderate
Schoofs, K. et al.	2	0	0	2/9	Low
Ococks E. et al. (Ann Oncol, 2021)	3	2	2	7/9	High
Ococks E. et al. (Gastroenterology, 2021)	3	0	2	5/9	Moderate
Hofste L.S.M. et al.	4	2	3	9/9	High
Bai L. et al.	4	0	2	6/9	Moderate
Liu Z. et al.	3	2	3	8/9	High
Yuan S.Q. et al.	4	2	3	9/9	High
Zhou H. et al.	2	0	2	4/9	Moderate
Yang J. et al.	3	2	3	8/9	High
Leal A. et al.	3	0	3	6/9	Moderate
Kim Y.W. et al.	3	0	2	5/9	Moderate
Cabel L. et al.	3	0	3	6/9	Moderate
Zaanan A. et al.	4	2	3	9/9	High
Hu Q. et al.	3	1	1	5/9	Moderate
Iden C.R. et al.	4	1	3	8/9	High
Lander E.M. et al.	3	0	3	6/9	Moderate
Huffman B.M. et al.	3	2	2	7/9	High

Supplementary Table S1: Quality assessment of the 27 included studies using the Newcastle-Ottawa Scale (NOS) for cohort studies. Each study was evaluated across three domains: Selection (maximum 4 stars), Comparability (maximum 2 stars), and Outcome (maximum 3 stars), yielding a maximum possible score of 9. Studies scoring 7–9 were classified as high quality, 4–6 as moderate quality, and 0–3 as low quality. Total NOS scores ranged from 2 to 9, with 14 of 27 studies classified as high quality, 12 as moderate quality, and 1 (a proof-of-concept methylation feasibility study) as low quality. The most common limitations were small sample size, single-center design, retrospective design in several cohorts, and heterogeneity in ctDNA detection platforms and sampling timepoints.
